# Development and retrospective evaluation of a clinical decision support system for the efficient detection of drug-related problems by clinical pharmacists

**DOI:** 10.1007/s11096-022-01505-5

**Published:** 2022-12-14

**Authors:** Christian Skalafouris, Anne-Laure Blanc, Olivier Grosgurin, Christophe Marti, Caroline Samer, Christian Lovis, Pascal Bonnabry, Bertrand Guignard

**Affiliations:** 1 Pharmacy, Geneva University Hospitals, Rue Gabrielle-Perret-Gentil 4, 1205 Geneva, Switzerland; 2 Institute of Pharmaceutical Sciences of Western Switzerland (ISPSO), School of Pharmaceutical Sciences, University of Geneva, Geneva, Switzerland; 3 Pharmacy of the Eastern Vaud Hospitals, Route du Vieux Séquoia 20, 1847 Rennaz, Switzerland; 4 General Internal Medicine Division, Geneva University Hospitals, Rue Gabrielle-Perret-Gentil 4, 1205 Geneva, Switzerland; 5 Clinical Pharmacology and Toxicology Division, Geneva University Hospitals, Rue Gabrielle-Perret-Gentil 4, 1205 Geneva, Switzerland; 6 Division of Medical Information Sciences, Geneva University Hospitals, Rue Gabrielle-Perret-Gentil 4, 1205 Geneva, Switzerland

**Keywords:** Clinical decision support system, Clinical pharmacy, Medication review, Rule-based system

## Abstract

**Background:**

Clinical decision support systems (CDSS) can help identify drug-related problems (DRPs). However, the alert specificity remains variable. Defining more relevant alerts for detecting DRPs would improve CDSS.

**Aim:**

Develop electronic queries that assist pharmacists in conducting medication reviews and an assessment of the performance of this model to detect DRPs.

**Method:**

Electronic queries were set up in CDSS using “triggers” from electronic health records: drug prescriptions, laboratory values, medical problems, vital signs, demographics. They were based on a previous study where 315 patients admitted in internal medicine benefited from a multidisciplinary medication review (gold-standard) to highlight potential DRPs. Electronic queries were retrospectively tested to assess performance in detecting DRPs revealed with gold-standard. For each electronic query, sensitivity, specificity, positive and negative predictive value were computed.

**Results:**

Of 909 DRPs, 700 (77.8%) were used to create 366 electronic queries. Electronic queries correctly detected 77.1% of DRPs, median sensitivity and specificity reached 100.0% (IQRs, 100.0%–100.0%) and 99.7% (IQRs, 97.0%–100.0%); median positive predictive value and negative predictive value reached 50.0% (IQRs, 12.5%–100.0%) and 100.0% (IQRs, 100.0%–100.0%). Performances varied according to “triggers” (*p* < 0.001, best performance in terms of predictive positive value when exclusively involving drug prescriptions).

**Conclusion:**

Electronic queries based on electronic heath records had high sensitivity and negative predictive value and acceptable specificity and positive predictive value and may contribute to facilitate medication review. Implementing some of these electronic queries (the most effective and clinically relevant) in current practice will allow a better assessment of their impact on the efficiency of the clinical pharmacist.

**Supplementary Information:**

The online version contains supplementary material available at 10.1007/s11096-022-01505-5.

## Impact statements

- Pharmaceutical interventions performed during the medication review can feed CDSS in order to increase clinical pharmacists efficiency in detecting drug related problem
- Using such a CDSS is only relevant if the alerts are associated with an acceptable positive predictive value (number of truly positive alerts to the total number of alerts)
- Alerts with a low positive predictive value should otherwise be considered–for particularly risky situations–if clinical pharamacists can allocate time to contextualize alerts

## Introduction

Clinical decision support systems (CDSS) are important for safe medication management. Systems based on computerized physician order entry (CPOE) can generate reminders (e.g., measure an antibiotic’s blood level after a certain interval) and alerts (e.g., drug interactions or dosages unsuited to pathophysiological conditions) aimed at preventing adverse drug events (ADEs) [1]. These systems’ principal benefit is that they facilitate physician adherence to appropriate care guidelines or medical practices, including prescribing guidelines [2]. Although some alerts are correlated with risk situations, many others lack specificity (Sp) with a large number of false positives or irrelevant alerts while disrupting the workflow excessively. This phenomenon of over-alerting leads to alert fatigue, which results in poor clinician compliance [3]. Alert fatigue is characterised as a poor signal-to-noise ratio caused by CDSS issuing interruptive alerts. It is "mental fatigue experienced by healthcare professionals who are confronted with numerous alerts and reminders from the use of CDSS" [4]. Alert fatigue causes physicians to ignore 49–96% of alerts [5].

One solution to alert fatigue is to distribute CDSS-mediated monitoring points among health professionals by promoting the contextualization of alerts. Indeed, many teams use CDSS designed for clinical pharmacists as part of their medication review processes [6–9]. Such tools are used in routine screening for specific drug-related problems (DRPs); they supplement the CDSS intended for physicians, which are targeted specifically at the drug prescription phase [6–9]. Alerts contextualized by clinical pharmacists and then communicated to prescribers seem to effectively highlight the riskiest situations and lead to optimized drug therapy: prescribers’ acceptance rates of pharmacists’ suggestions range from 63.0 to 83.0% [6, 10, 11]. However, these approaches do not solve the problem of futile alerts (providing a signal that is not relevant to the patient's clinical/biological context). Studies described proportions of alerts leading to pharmacists’ intervention varying from 8.0 to 51.0%.

Better performance is expected as patients’ electronic health records (EHRs) become more structured. These varied data (laboratory results, demographic data, prescriptions, radiology reports and images, admission and follow-up notes, diagnostic codes) are hosted in clinical data warehouses [12]. Archived data can be reused retrospectively, beyond the production of direct clinical care, in the context of research and quality improvement studies [13]. Some data warehouses also support CDSS (e.g. by allowing the estimation of risk factors or the calculation of predictive scores) from hosted data processed with electronic queries (EQs) thus triggering alerts. Indeed, the prospects offered by the exploitation of clinical data warehouses in clinical practice are particularly interesting. At the University Hospitals of Geneva, Switzerland, we have developed our own CDSS called PharmaCheck which is managed by the pharmacy department. PharmaCheck uses data from the clinical data warehouse to produce alerts for the clinical pharmacist. Currently, some twenty EQs are used to target DRPs associated to a high risk of iatrogeny [14]. Nevertheless, the use of this tool could be extended to identify more DRPs and facilitate medication review. Selecting which DRPs should be targeted using CDSS like PharmaCheck remains essential to increase clinical pharmacists efficiency in medication reviews.

### Aim

Develop EQs that assist pharmacists in conducting medication reviews and an assessment of the performance of this model to detect DRPs.

### Ethics approval

Study protocol was reviewed by the Canton of Geneva’s Ethics Committee, Switzerland (Req-2017–00988). The Committee decided that there was no need to go into the matter as this quality-improvement study was set up as standard practice not falling within the scope of the Swiss law on research on human beings. The project was part of quality improvement of care process without the objective of being a scientific research on human diseases or on the structure and functioning of the human body.

## Method

### Study design

In a previous study conducted from September to October 2015, an expert panel (one clinical pharmacist, one clinical pharmacologist, two senior hospital internists) identified 909 potential DRPs for 315 inpatients 48 h after admission to an internal medicine ward [9]. In the present study, the clinical reasoning that led to the detection of DRPs was dematerialized in the form of EQs based on triggers and implemented within our CDSS dedicated to clinical pharmacists. EQs were retrospectively run through the EHRs (hosted in clinical data warehouse) of the initial study’s patients. We assessed how well the EQs detected potential DRPs against the first study’s gold-standard expert panel detection method.

### Settings

In 2000-bed Geneva University Hospital, Switzerland, CPOE is supported by a CDSS dedicated to physicians for suggesting on-label, default dosages and routes of administration for each drug [15]. The CDSS also performs several checking procedures (e.g. medication duplication, drug–drug interactions). Primary care information systems are not connected to our hospital information system. PharmaCheck is another CDSS developed by and dedicated to our clinical pharmacy department [14]. PharmaCheck screens 20 high-risk situations and triggers alerts intended for clinical pharmacists who assess if recommending a treatment adjustment via a phonecall to prescribing physician is necessary. Our clinical pharmacists also provide medication reviews, making treatment optimization suggestions during medical rounds. The situations they detect may be less critical than those detected by PharmaCheck’s overall electronic screening. Treatment optimization suggestions are very patient-centered but time-consuming activity, only available for approximately 20% of the 200 patients admitted in general internal medicine and acute geriatrics respectively, because of the lack of clinical pharmacists (with 5 pharmacists participating in medical rounds). Thus, using PharmaCheck was a strategy to facilitate and accelerate the detection of DRPs.

### Selecting DRPs and developing electronic queries

We considered the medication reviews performed by our previous study’s expert multidisciplinary panel to be the gold-standard approach [9]. This medication review was proceed remotely, based on computerised medical records, without contact with patients and the medical staff. Thus, our new EQs resulted from analyzing the 909 DRPs detected by that panel. DRPs were selected if they were convertible into clinical rules within PharmaCheck in the form of EQs. They had to contain structured data in patients’ EHRs: drug prescriptions using the Anatomical Therapeutic Chemical (ATC) classification, laboratory values, vital signs, or demographics. Although medical problems are now recorded as structured data within our EHRs, they were not when the 315 patients from our previous study were admitted. Thus, these free-text terms had to be manually converted into the structured codes of the International Classification of Diseases 10^th^ revision (ICD-10) using a correspondence table built by our institution’s Medical Information Sciences Division [16]. DRPs involving drug omission at admission were not selected for inclusion as treatments taken at home were not documented as structured data in EHRs.

For example, some DRPs identified by the expert panel were associated with an increased risk of constipation in the presence of opioid treatments without laxatives and in absence of stools for at least 24 h. The conversion of this clinical reasoning into EQ was structured as follow. The simultaneous respect of these conditions leads to the activation of an alert:

- Prescription of constipation-providing medication identified via ATC code (e.g., prescription of N02AE01–buprenorphine or N07BC02–methadone or N02AA01–morphine).
- No concurrent prescription of laxative therapy (ATC code: A06–drugs for constipation).
- Absence of stools during the last 24 h identified via structured patient value ("pv.faeces = 0").

DRPs were classified according to a national frame of reference [9]. Six categories of EQs were distinguished based on: drug information exclusively; drug information + medical problems; drug information + laboratory values; drug information + vital signs; drug information + demographic data; complex EQs. EQs set for the first five categories used two triggers (e.g. ATC + dose; ATC + ICD-10 code; ATC + laboratory value), complex EQs category used more than two triggers (e.g., ATC + dose + ICD-10 code).

### Detecting DRPs using electronic queries and assessing performance

EQs were retrospectively tested in previous study’s 315 patients EHRs (day by day for the 2 months study period). We distinguished:

- True positives (TPs): EQ detected a DRP in patient’s EHR, matching the gold-standard
- False positives (FPs): EQ detected a DRP in patient’s EHR, contradicting the gold-standard
- True negatives (TNs): EQ did not detect a DRP in patient’s EHR, matching the gold-standard
- False negatives (FNs): EQ did not detect a DRP in patient’s EHR, contradicting the gold-standard

**Fig. 1:**
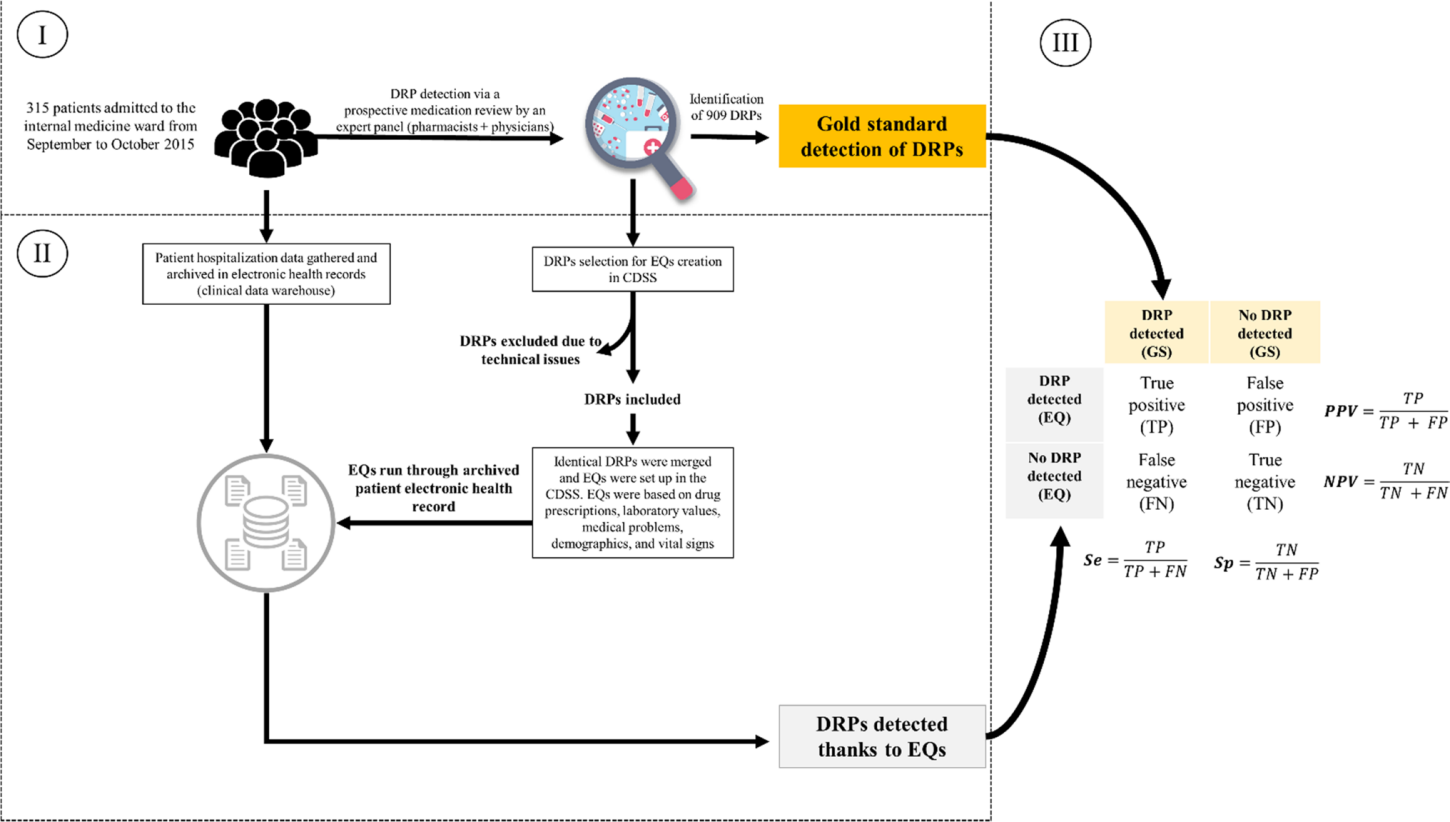
The study method’s three steps. I—*Definition of the gold standard* (GS): Based on a previous study of 315 patients, medication reviews carried out by an expert panel (a clinical pharmacist, a clinical pharmacologist, and two internists) were considered the GS detection method for drug-related problems (DRPs) [7]. Over two months, 909 potential DRPs were detected. II—*Analysis of drug therapy problems and setting up electronic queries*: All the DRPs detected using the GS approach were reviewed by a clinical pharmacist. All those that could be detected automatically using electronic queries (EQs) were selected for inclusion. EQs had to be based on structured triggers according to international categorizations (e.g., ICD-10, ATC code) or local nomenclatures. EQs were then set up within our clinical decision support system (CDSS) and used to retrospectively check through our clinical data warehouse for hospitalization data concerning our previously admitted patients. As patients’ medical problems were, at that time, expressed in free text in their EHR, these were manually coded according to ICD-10 nomenclature so that these data could be matched with the EQs. III—*Comparing DRP detection using EQs and the GS*: The performance of EQs was evaluated by measuring the number of true-positive (the GS had also identified a DRP) and false-positive (the EQ had detected a DRP but the GS had not) alerts. Also, we measured true-negative (the absence of an alert, neither method detected a DRP) and false-negative alerts (absence of an EQ alert when the GS had detected a DRP). Each EQ was thus associated with a sensitivity (Se), a specificity (Sp), a positive predictive value (PPV), and a negative predictive value (NPV)

Each alert was counted once, even if it was triggered multiple times during analysis. We calculated the sensitivity $\left( {Se = \frac{TP}{{TP + FN}}} \right)$ and specificity $\left( {Sp = \frac{TN}{{TN + FP}}} \right)$ of EQs as well as their positive predictive value $\left( {PPV = \frac{TP}{{TP + FP}}} \right)$ and negative predictive value $\left( {NPV = \frac{TN}{{TN{ } + FN}}} \right)$ [17]. DRPs prevalence (Pr) was determined by calculating their occurrence out of the total number of patients included. Figure 1 illustrates method main steps.

Our study focuses on the purely "technical" aspect, i.e. the evaluation of an electronic model in the detection of DRPs. We will not evaluate here the "clinical" aspect, i.e. the relevance of the risk situations identified and targeted by our EQs. Indeed, some of the situations identified in 2015 would be potentially inappropriate given the evolution of practices and evidence-based knowledge (some practices considered risky in 2015 could be acceptable in 2022–some medications associated with DRPs may no longer be referenced in drug formulary).

### Data analysis

Se, Sp, PPV, and NPV were calculated for each EQ. As data were non-normally distributed, the results aggregating all the EQs were described using medians, interquartile ranges, and minimum and maximum values: median (IQR; min–max). The median DRP prevalence was calculated by taking into account the prevalence of each DRP targeted by an EQ. EQs were listed according to the six categories of triggers, and a Kruskal–Wallis test (*p* ≤ 0.001) was used to examine the differences in Se, Sp, PPV, and NPV between each category. Data analyses were conducted using R software (version 3.6.3).

## Results

The expert panel detected 700 DRPs (77.8%) suitable for the development of clinical rules, involving 97.8% (n = 308) of the 315 patients included; 209 DRPs (22.2%) could not be translated into EQs, as they were involving a break in continuity of care (medication taken at home but not renewed at admission). The distribution of different categories of DRPs is presented in Table 1. DRPs identified multiple times were counted only once, resulting in 366 different DRPs and a corresponding number of EQs (DRPs are described in the Supplementary material). Most DRPs (290; 79.2%) were unique to one patient, but 76 (20.8%) concerned at least 2 patients. Median DRP prevalence was of 0.32% (IQR: 0.32%–0.32%; 0.32%–23.5%).

**Table 1:** Distribution of drug-related problems targeted by electronic queries

Drug-related problem type	*n* (%)
Drug–drug interaction	112 (30.6%)
Over-prescription—duplicate therapy	77 (21.0%)
Adverse drug events	52 (14.2%)
Untreated indication/non-compliance with guidelines	44 (12.0%)
Inadequate dosage for physiological state	23 (6.3%)
Underdosage	17 (4.6%)
Inappropriate monitoring	13 (3.6%)
Inappropriate route of administration or galenic formulation	10 (2.7%)
Inappropriate time or frequency of administration	10 (2.7%)
Overdosage	6 (1.6%)
Inappropriate treatment duration	2 (0.5%)
Total	366 (100.0%)

The 366 EQs ran through previous study’s 315 patients EHR resulted in 4,051 alerts with 540 TPs (13.3%) and 3,511 FPs (86.7%). Thus, EQs identified 77.1% of DRPs identified using the gold-standard detection method (540 TPs out of 700 DRPs reported).

Concerning positive alerts (DRPs detected in patients’ EHRs), EQs were associated with a median of 1 TP (IQR: 1–1; min–max: 0–54) and 1 FP (IQR: 0–9; min–max: 0–189). Concerning negative alerts (no DRPs detected in patients’ EHRs), EQs were associated with a median of 306 TN alerts (IQR: 297–307; min–max: 118–307) and 0 FN alerts (IQR: 0–0; min–max: 0–20).

Compared to the gold-standard DRP detection method, detection via EQs was associated with a median Se of 100% (IQR: 100.0%–100.0%; min–max: 0.0%–100.0%) and a median Sp of 99.7% (IQR: 97.1%–100.0%; min–max: 38.4%–100.0%). Moreover, median PPV was 50.0% (IQR: 12.5%–100.0%; min–max: 0.0%–100.0%) and median NPV was 100% (IQR: 100.0%–100.0%; min–max: 88.6%–100.0%).

A significant difference was observed in the median values of Se, Sp, PPV, and NPV depending on the triggers involved in the EQs set up (*p* < 0.001). These are represented in Table 2.

**Table 2:** Performance of DRP detection by electronic queries according to triggers

Electronic query involving	Number of EQs	Median IQR; min–max				
		Se	Sp	PPV	NPV	Pr
Drug prescription	168 (46.2%)	100.0% IQR: 100.0%–100.0% min–max: 0.0%–100.0%	100% IQR: 99.7%–100.0% min–max: 80.0%–100.0%	100% IQR: 33.5%–100.0% min–max: 0.0%–100.0%	100% IQR: 100.0%–100.0% min–max: 97.5%–100.0%	0.32% IQR: 0.32%–0.63% min–max: 0.32%–23.5%
Drug prescription + medical problems	112 (30.6%)	100.0% IQR: 100.0%–100.0% min–max: 0.0%–100.0%	96.1% IQR: 96.0%–99.7% min–max: 57.7%–100.0%	25.0% IQR: 8.3%–50.0% min–max: 0.0%–100.0%	100.0% IQR: 100.0%–100.0% min–max: 88.6%–100.0%	0.32% IQR: 0.32%–0.32% min–max: 0.32%–5.7%
Drug prescription + laboratory values	51 (13.7%)	100.0% IQR: 77.5%–100.0% min–max: 0.0%–100.0%	98.4% IQR: 93.4%–99.7% min–max: 41.5%–100.0%	14.6% IQR: 6.0%–50.0% min–max: 0.0%–100.0%	100.0% IQR: 99.8%–100.0% min–max: 95.3%–100.0%	0.32% IQR: 0.32%–0.32% min–max: 0.32%–6.7%
Drug prescription + vital signs	11 (3.0%)	100.0% IQR: 66.7%–100.0% min–max: 0.0%–100.0%	99.4% IQR: 87.8%–99.4% min–max: 38.5%–100.0%	3.1% IQR: 0.8%–33.3% min–max: 0.0%–33.3%	100.0% IQR: 99.8%–100.0% min–max: 99.3%–100.0%	0.32% IQR: 0.32%–0.32% min–max: 0.32%–1.3%
Drug prescription + demographic data	6 (1.6%)	100.0% IQR: 100.0%–100.0% min–max: 40.0%–100.0%	99.8% IQR: 97.4%–100.0% min–max: 94.5%–100.0%	75.0% IQR: 25.0%–100.0% min–max: 5.6%–100.0%	100.0% IQR: 100.0%–100.0% min–max: 99.0%–100.0%	0.32% IQR: 0.32%–0.32% min–max: 0.32%–0.63%
Complex queries	18 (4.9%)	100% IQR: 57.6%–100.0% min–max: 0.0%–100.0%	97.2% IQR: 94.2%–99.5% min–max: 83.0%–100.0%	9.5% IQR: 2.4%–45.5% min–max: 0.0%–100.0%	100.0% IQR: 99.7%–100.0% min–max: 98.2%–100.0%	0.32% IQR: 0.32%–0.56% min–max: 0.32%–5.1%
Total	366 (100%)	100.0% IQR: 100.0%–100.0% min–max: 0.0%–100.0%	99.7% IQR: 97.0%–100.0% min–max: 38.4%–100.0%	50.0% IQR: 12.5%–100.0% min–max: 0.0%–100.0%	100.0% IQR: 100.0%–100.0% min–max: 88.6%–100.0%	0.32% IQR: 0.32%–0.32% min–max: 0.32%–23.5%

## Discussion

### Statement of key findings

In this project, we created 366 new EQs to enrich our PharmaCheck CDSS, originally intended to detect just 20 high-risk clinical situations related to drugs. The EQs’ ability to detect DRPs was retrospectively assessed in comparison to a gold-standard medication review performed by an expert panel. EQs detected 77.1% of the DRPs detected using the gold-standard method, and they demonstrated high Se, Sp, NPV while PPV varied significantly by type of triggers involved in the EQs.

### Strengths and weaknesses

Our study had some strengths. First, considering technical criteria, we succeeded in creating EQs that detected 77.8% of the DRPs detected using the gold-standard method. We assessed EQs’ performance against a validated multidisciplinary medication review. This comparison allowed us to calculate each EQ’s Se (probability that the EQ will alert us to a potential DRP), Sp (probability that the EQ will not alert us to the absence of a DRP), NPV (probability that there is no DRP when there is no alert), and PPV (probability that there is a DRP when there is an alert). Thus, the performance of each EQ could be retrospectively measured to determine which would be the most relevant for prospective use.

Our study also had some limitations. Inclusion criteria depended on data availability and DRPs involving treatment discontinuity between home and hospital were excluded. The choice of the gold-standard was an other limitation. We considered expert panel opinion to be a reference for detecting DRPs; therefore, any DRP detected by the EQs but not highlighted by the panel was considered as a FP. Yet, a CDSS can sometimes identify problems missed by experts [7]. In fact, we didn’t determine whether the FP were background noise or just situations not intercepted by experts. Second, expert panel reviewed medication remotely with a limited identification of some contextualizing elements that would have been useful to identify even more relevant DRPs. Finaly, EQs were tested retrospectively, without prospective validation on a new cohort of patients. Redefining our gold-standard, analysing EQ performance against a pharmacists prospective medication review (conduted inward, in contact with the patient and the medical/healthcare team) should improve our model.

### Interpretation

Carli et al.’s literature review revealed disparate Se (from 38.0–91.0%) and Sp (from 11.0–96.0%), which seemed to depend on the medical specialty or therapeutic class examined [18]. Nevertheless our EQs resulted in good overall Se and Sp, with median values of 100.0% and 99.7%, respectively. These values contrast with the high proportion of FPs (86.7%) since a fraction of alerts were corresponding to experts opinion (TPs = 13.3%). FPs are considered in the calculation of Sp, which also includes the number of TNs: $\left( {Sp = \frac{TN}{{TN + FP}}} \right)$. Thus, the apparently good Sp values considered a non-homogeneous distribution of FPs across the EQs, with most associated with a limited number of FPs, and some with a large number. Thus, 20 EQs produced 50% of FPs alerts. PPV relates the number of truly positive alerts to the total number of alerts (true positive and false positive). In this way it is possible to estimate, for each EQ, the fraction of significant alerts out of the number of alerts produced. As an example, those 20 queries that produced a maximum of false positives were associated with a PPV ranging from 0.5 to 8.0% (with one particular case-Treatment without clear indication exposing to a side effect (esomeprazole)-where the PPV is 40.0%).

With a view to routine use, EQs must perform to an acceptable level, particularly their NPV and PPV. Evaluating desired NPVs and PPVs should consider several criteria, such as their benefits, risks, and, more generally, the ability to accept or not the presence of FP or FN alerts [19]. PPVs vary widely (ranging from 8.0–83.0%) depending on the type of CDSS, and they perform better when they consider information describing the clinical context [18]. This is consistent with the values found in our study, where median PPV reached 50.0%. Few studies have assessed the NPV of CDSS similar to ours; however, the total NPV was comparable (90.0%) in a retrospective study assessing correlations between electronic alerts and the corresponding symptoms experienced by patients [20]. Furthermore, these values are largely influenced by prevalence (even for the best screening tests, PPV decreases when prevalence is low, and NPV increases inversely) [21].

We observed a significant difference in EQs’ performance in detecting DRPs according to the nature of the triggers. In 46.2% of cases, EQs based on data regarding drug prescriptions exclusively were the most efficient at predicting the presence of a DRP (easily characterizable since drug prescriptions are fully structured). In 48.6% of cases, EQs based on data related to drug prescriptions associated with clinical or laboratory values presented important variability in DRPs prediction (median PPVs ranging from 3.1–75.0%). Most of them used medical problems characterized with ICD-10 nomenclature (median PPV = 25.0%). Although medical problems were structured in patients’ EHRs a posteriori, using a correspondence table, using this nomenclature can be imprecise. Initial free-text descriptions of medical problems in EHRs were subject to variability, making ICD-10 code mapping complex [22]. Thus, an EQ may not be able to correctly identify a poorly coded medical problem if it has been set to detect a strict selection of ICD-10 codes. Other EQs used laboratory values (median PPV = 14.1%) or vital signs (median PPV = 3.1%) referring to quantifiable data using threshold values to trigger alerts. Taken individually, these values may not be accurate enough. Indeed, any rapid alteration of a vital parameter may require adapting medication, even if a threshold value has not been reached. Moreover values for individual patients may also be different (e.g. some medications require specific dose adjustments depending on the degree of renal function [23]). Eighteen DRPs required the creation of complex EQs involving more than two triggers. In principle, more triggers should better characterise a complex clinical situation, but then it is sufficient for just one of the conditions not to be fulfilled for an alert to be cancelled. This is even more problematic because the informational quality of each trigger can vary, as mentioned above.

### Further research

Detecting DRPs remains a time-consuming activity, and well-selected EQs could reduce this workload [6–8, 24]. In their model, Falconer et al. developed a patient prioritization system to achieve more efficient medication reviews [25]. This prioritization considered a risk score associated with electronic alerts. Associating a score to each EQ, would be an interesting option for optimizing our model: DRPs targeted by the EQs could be scored according to different dimensions (e.g. according to severity, frequency, avoidability of the clinical consequence). Thus, each patient could be associated with a total risk score so as to identify those for whom medication review is a priority. Criteria for selecting candidate EQs for inclusion in our CDSS in routine practice should also consider PPVs and NPVs. We noted that a minority of EQs was associated with a maximum number of FPs, which affected the Sp and PPV of the alerts, which decreased proportionally. Thus, it seems wise to retain the most efficient EQs—those with the maximum efficiency values. A first approach would be to decide on a threshold value of positive predictive value that is acceptable and compatible with the routine use of EQs. A second approach would concern clinical situations which it would be relevant to screen using a CDSS but for which the performance in terms of PPV is not yet acceptable. Here, the associated queries would have to be improved by redefining, for example, the criteria for triggering alerts.

## Conclusion

The EQs we developed are directly inspired by expert practice, and allowed retrospective identification of DRPs in patients EHRs. The queries associated with the best performances in terms of PPV would probably be useful for an automated detection of potential DRPs, in routine practice and prospectively. Nevertheless, some EQs produced potential inappropriate signals associated with a large number of false positives (likely to expose clinical pharmacists to alert fatigue). It will therefore be important to carefully select and optimize these unsatisfactory queries that target the most critical DRPs. In this context, prospective studies seem relevant to determine which alerts are clinically relevant during medication reviews. In future, the identification of DRPs eligible for automated detection, in close collaboration with clinical pharmacists (by taking into account their needs and wishes), offers a perspective of individualized decision support. Pharmacists could use this aid, consistent with their practice, to save time when preparing their visit in wards. Moreover, such electronic queries could be shared within pharmacists and contribute to the standardization of medication review.

## Supplementary Information

Below is the link to the electronic supplementary material.

Supplementary file1 (DOCX 115 KB)
