# Supplementary material for: Development and retrospective evaluation of a clinical decision support system for the efficient detection of drug-related problems by clinical pharmacists
Source: Int J Clin Pharm. 2022 Dec 14;45(2):406–13. doi: 10.1007/s11096-022-01505-5 (PMC10147748; doi:10.1007/s11096-022-01505-5)
Supplement: Supplementary file 1 — Supplementary file1 (DOCX 115 KB) [file 11096_2022_1505_MOESM1_ESM.docx]

# Electronic Supplementary Material

| **Triggers** | **DRP classification** | **DRP targeted** | **TP** | **FP** | **TN** | **FN** | **Se** | **Sp** | **VPP** | **VPN** |
| --- | --- | --- | --- | --- | --- | --- | --- | --- | --- | --- |
| Drug | Drug-drug interaction | Anticholinegic side effects (scopolamine + glycopyrronium) | 0 | 0 | 307 | 1 | 0.0% | 100.0% | 0.0% | 99.7% |
| Drug | Drug-drug interaction | Decreased therapeutic effect (amiodarone + clopidogrel) | 1 | 1 | 306 | 0 | 100.0% | 99.7% | 50.0% | 100.0% |
| Drug | Drug-drug interaction | Decreased therapeutic effect (atorvastatine + magaldrate) | 1 | 0 | 307 | 0 | 100.0% | 100.0% | 100.0% | 100.0% |
| Drug | Drug-drug interaction | Decreased therapeutic effect (atorvastatine + rifampicine) | 1 | 0 | 307 | 0 | 100.0% | 100.0% | 100.0% | 100.0% |
| Drug | Drug-drug interaction | Decreased therapeutic effect (ciprofloxacine + magaldrate) | 1 | 0 | 307 | 0 | 100.0% | 100.0% | 100.0% | 100.0% |
| Drug | Drug-drug interaction | Decreased therapeutic effect (ciprofloxacine + magnesium chloride) | 1 | 0 | 306 | 1 | 50.0% | 100.0% | 100.0% | 99.7% |
| Drug | Drug-drug interaction | Decreased therapeutic effect (clopidogrel + ciprofloxacine) | 1 | 0 | 307 | 0 | 100.0% | 100.0% | 100.0% | 100.0% |
| Drug | Drug-drug interaction | Decreased therapeutic effect (clopidogrel + esomeprazole) | 2 | 9 | 296 | 1 | 66.7% | 97.0% | 18.2% | 99.7% |
| Drug | Drug-drug interaction | Decreased therapeutic effect (clopidogrel + nifedipine + sertraline) | 0 | 1 | 307 | 0 | 0.0% | 99.7% | 0.0% | 100.0% |
| Drug | Drug-drug interaction | Decreased therapeutic effect (duloxetine + metoprolol) | 1 | 1 | 306 | 0 | 100.0% | 99.7% | 50.0% | 100.0% |
| Drug | Drug-drug interaction | Decreased therapeutic effect (glibenclamide + amiodarone) | 1 | 0 | 307 | 0 | 100.0% | 100.0% | 100.0% | 100.0% |
| Drug | Adverse drug events | Decreased therapeutic effect (indacaterol + propranolol) | 1 | 0 | 307 | 0 | 100.0% | 100.0% | 100.0% | 100.0% |
| Drug | Drug-drug interaction | Decreased therapeutic effect (irbesartan + dexamethasone) | 1 | 0 | 307 | 0 | 100.0% | 100.0% | 100.0% | 100.0% |
| Drug | Drug-drug interaction | Decreased therapeutic effect (levofloxacine + human insulin) | 1 | 1 | 306 | 0 | 100.0% | 99.7% | 50.0% | 100.0% |
| Drug | Drug-drug interaction | Decreased therapeutic effect (levofloxacine + magnesium ) | 1 | 2 | 305 | 0 | 100.0% | 99.3% | 33.3% | 100.0% |
| Drug | Drug-drug interaction | Decreased therapeutic effect (levofloxacine + sodium bicarbonate) | 1 | 0 | 307 | 0 | 100.0% | 100.0% | 100.0% | 100.0% |
| Drug | Drug-drug interaction | Decreased therapeutic effect (levothyroxine (T4) + ciprofloxacine) | 1 | 0 | 307 | 0 | 100.0% | 100.0% | 100.0% | 100.0% |
| Drug | Drug-drug interaction | Decreased therapeutic effect (levothyroxine (T4) + magaldrate) | 1 | 0 | 307 | 0 | 100.0% | 100.0% | 100.0% | 100.0% |
| Drug | Drug-drug interaction | Decreased therapeutic effect (magaldrate + allopurinol + rosuvastatine) | 1 | 0 | 307 | 0 | 100.0% | 100.0% | 100.0% | 100.0% |
| Drug | Drug-drug interaction | Decreased therapeutic effect (mycophenolate mofetil + esomeprazole) | 1 | 1 | 306 | 0 | 100.0% | 99.7% | 50.0% | 100.0% |
| Drug | Drug-drug interaction | Decreased therapeutic effect (mycophenolate mofetil + levofloxacine) | 1 | 0 | 307 | 0 | 100.0% | 100.0% | 100.0% | 100.0% |
| Drug | Drug-drug interaction | Decreased therapeutic effect (oxycodone + oxycodone) | 1 | 0 | 307 | 0 | 100.0% | 100.0% | 100.0% | 100.0% |
| Drug | Drug-drug interaction | Decreased therapeutic effect (oxycodone + venlafaxine) | 1 | 1 | 306 | 0 | 100.0% | 99.7% | 50.0% | 100.0% |
| Drug | Drug-drug interaction | Decreased therapeutic effect (rivaroxaban + duloxetine) | 1 | 2 | 305 | 0 | 100.0% | 99.3% | 33.3% | 100.0% |
| Drug | Drug-drug interaction | Decreased therapeutic effect (ticagrelor + phenobarbital) | 1 | 0 | 307 | 0 | 100.0% | 100.0% | 100.0% | 100.0% |
| Drug | Drug-drug interaction | Decreased therapeutic effect (tramadol + dexamethasone) | 1 | 0 | 307 | 0 | 100.0% | 100.0% | 100.0% | 100.0% |
| Drug | Drug-drug interaction | Decreased therapeutic effect (valproic acid + imipenem + cilastatine) | 1 | 0 | 307 | 0 | 100.0% | 100.0% | 100.0% | 100.0% |
| Drug | Drug-drug interaction | Decreased therapeutic effect (venlafaxine + rifampicine) | 1 | 0 | 307 | 0 | 100.0% | 100.0% | 100.0% | 100.0% |
| Drug | Drug-drug interaction | Decreased therapeutic effect (zolpidem + rifampicine) | 1 | 0 | 307 | 0 | 100.0% | 100.0% | 100.0% | 100.0% |
| Drug | Drug-drug interaction | Decreased therapeutic effect sous-osage prednisone (prednisone + magaldrate ) | 1 | 2 | 305 | 0 | 100.0% | 99.3% | 33.3% | 100.0% |
| Drug | Drug-drug interaction | Drug accumulation exposig to various side effects (acenocoumarol + citalopram) | 1 | 1 | 306 | 0 | 100.0% | 99.7% | 50.0% | 100.0% |
| Drug | Drug-drug interaction | Drug accumulation exposig to various side effects (acenocoumarol + sertraline) | 1 | 1 | 306 | 0 | 100.0% | 99.7% | 50.0% | 100.0% |
| Drug | Drug-drug interaction | Drug accumulation exposig to various side effects (acetylsalicilic acid + citalopram) | 1 | 1 | 306 | 0 | 100.0% | 99.7% | 50.0% | 100.0% |
| Drug | Drug-drug interaction | Drug accumulation exposig to various side effects (amlodipine + clarithromycine) | 1 | 1 | 306 | 0 | 100.0% | 99.7% | 50.0% | 100.0% |
| Drug | Drug-drug interaction | Drug accumulation exposig to various side effects (amlodipine + clopidogrel + esomeprazole) | 1 | 2 | 305 | 0 | 100.0% | 99.3% | 33.3% | 100.0% |
| Drug | Drug-drug interaction | Drug accumulation exposig to various side effects (atorvastatine + primidone + amiodarone) | 1 | 0 | 307 | 0 | 100.0% | 100.0% | 100.0% | 100.0% |
| Drug | Drug-drug interaction | Drug accumulation exposig to various side effects (atorvastatine + amiodarone) | 1 | 1 | 306 | 0 | 100.0% | 99.7% | 50.0% | 100.0% |
| Drug | Drug-drug interaction | Drug accumulation exposig to various side effects (atorvastatine) | 1 | 3 | 304 | 0 | 100.0% | 99.0% | 25.0% | 100.0% |
| Drug | Drug-drug interaction | Drug accumulation exposig to various side effects (ciclosporine + diltiazem) | 1 | 0 | 307 | 0 | 100.0% | 100.0% | 100.0% | 100.0% |
| Drug | Drug-drug interaction | Drug accumulation exposig to various side effects (clarithromycine + atorvastatine) | 1 | 2 | 305 | 0 | 100.0% | 99.3% | 33.3% | 100.0% |
| Drug | Drug-drug interaction | Drug accumulation exposig to various side effects (digoxine + clarithromycine) | 1 | 1 | 306 | 0 | 100.0% | 99.7% | 50.0% | 100.0% |
| Drug | Drug-drug interaction | Drug accumulation exposig to various side effects (escitalopram + esomeprazole) | 1 | 7 | 300 | 0 | 100.0% | 97.7% | 12.5% | 100.0% |
| Drug | Overprescription—duplicate therapy | Drug accumulation exposig to various side effects (indacaterol + salmeterol) | 1 | 0 | 307 | 0 | 100.0% | 100.0% | 100.0% | 100.0% |
| Drug | Drug-drug interaction | Drug accumulation exposig to various side effects (levofloxacine + probenecide) | 1 | 0 | 307 | 0 | 100.0% | 100.0% | 100.0% | 100.0% |
| Drug | Drug-drug interaction | Drug accumulation exposig to various side effects (lorazepam + verapamil) | 1 | 0 | 307 | 0 | 100.0% | 100.0% | 100.0% | 100.0% |
| Drug | Drug-drug interaction | Drug accumulation exposig to various side effects (methadone + tramadol) | 1 | 1 | 306 | 0 | 100.0% | 99.7% | 50.0% | 100.0% |
| Drug | Drug-drug interaction | Drug accumulation exposig to various side effects (metoprolol + fluoxetin) | 1 | 0 | 307 | 0 | 100.0% | 100.0% | 100.0% | 100.0% |
| Drug | Drug-drug interaction | Drug accumulation exposig to various side effects (paracetamol + rifampicine) | 2 | 0 | 305 | 1 | 66.7% | 100.0% | 100.0% | 99.7% |
| Drug | Drug-drug interaction | Drug accumulation exposig to various side effects (propranolol + metoclopramide) | 1 | 1 | 306 | 0 | 100.0% | 99.7% | 50.0% | 100.0% |
| Drug | Drug-drug interaction | Drug accumulation exposig to various side effects (rivaroxaban + amiodarone) | 1 | 1 | 306 | 0 | 100.0% | 99.7% | 50.0% | 100.0% |
| Drug | Drug-drug interaction | Drug accumulation exposig to various side effects (simvastatine + ciclosporine) | 0 | 0 | 307 | 1 | 0.0% | 100.0% | 0.0% | 99.7% |
| Drug | Drug-drug interaction | Drug accumulation exposig to various side effects (simvastatine + clarithromycine) | 1 | 3 | 304 | 0 | 100.0% | 99.0% | 25.0% | 100.0% |
| Drug | Drug-drug interaction | Drug accumulation exposig to various side effects (simvastatine + diltiazem) | 1 | 0 | 307 | 0 | 100.0% | 100.0% | 100.0% | 100.0% |
| Drug | Drug-drug interaction | Drug accumulation exposig to various side effects (venlafaxine + clarithromycine) | 1 | 0 | 307 | 0 | 100.0% | 100.0% | 100.0% | 100.0% |
| Drug | Adverse drug events | Drug accumulation exposig to various side effects (venlafaxine + trazodone) | 1 | 1 | 306 | 0 | 100.0% | 99.7% | 50.0% | 100.0% |
| Drug | Drug-drug interaction | Drug-induced central nervous system depression (alprazolam + flurazepam) | 1 | 0 | 307 | 0 | 100.0% | 100.0% | 100.0% | 100.0% |
| Drug | Drug-drug interaction | Drug-induced central nervous system depression (alprazolam + zolpidem) | 1 | 0 | 307 | 0 | 100.0% | 100.0% | 100.0% | 100.0% |
| Drug | Overprescription—duplicate therapy | Drug-induced central nervous system depression (bromazepam + lorazepam) | 1 | 0 | 307 | 0 | 100.0% | 100.0% | 100.0% | 100.0% |
| Drug | Overprescription—duplicate therapy | Drug-induced central nervous system depression (buprenorphine + tramadol) | 1 | 1 | 306 | 0 | 100.0% | 99.7% | 50.0% | 100.0% |
| Drug | Drug-drug interaction | Drug-induced central nervous system depression (clonazepam + buprenorphine + clarithromycine) | 1 | 0 | 307 | 0 | 100.0% | 100.0% | 100.0% | 100.0% |
| Drug | Drug-drug interaction | Drug-induced central nervous system depression (clorazepate + pregabaline + risperidone + mirtzazapine) | 1 | 0 | 307 | 0 | 100.0% | 100.0% | 100.0% | 100.0% |
| Drug | Untreated indication/non-conforming with guidelines | Drug-induced central nervous system depression (codeine+ morphine) | 1 | 0 | 307 | 0 | 100.0% | 100.0% | 100.0% | 100.0% |
| Drug | Drug-drug interaction | Drug-induced central nervous system depression (codeine+ morphine) (oxazepam + flurazepam) | 1 | 1 | 306 | 0 | 100.0% | 99.7% | 50.0% | 100.0% |
| Drug | Drug-drug interaction | Drug-induced central nervous system depression (codeine+ morphine) (oxazepam + zolpidem) | 1 | 3 | 304 | 0 | 100.0% | 99.0% | 25.0% | 100.0% |
| Drug | Drug-drug interaction | Drug-induced central nervous system depression (tapentadol + tramadol) | 1 | 0 | 307 | 0 | 100.0% | 100.0% | 100.0% | 100.0% |
| Drug | Drug-drug interaction | Drug-induced central nervous system depression(zolpidem + morphine + mirtazapine + oxazepam) | 1 | 0 | 307 | 0 | 100.0% | 100.0% | 100.0% | 100.0% |
| Drug | Drug-drug interaction | Drug-induced hyperkaliemia (canagliflozine + valsartan + enoxaparine) | 1 | 0 | 307 | 0 | 100.0% | 100.0% | 100.0% | 100.0% |
| Drug | Drug-drug interaction | Drug-induced hypotension (clarithromycine + tamsulosine+ losartan) | 1 | 0 | 307 | 0 | 100.0% | 100.0% | 100.0% | 100.0% |
| Drug | Overprescription—duplicate therapy | Drug-induced hypotension (lercanidipine + nifedipine) | 1 | 0 | 307 | 0 | 100.0% | 100.0% | 100.0% | 100.0% |
| Drug | Drug-drug interaction | Drug-induced lowering of epileptic threshold (paroxetine + tramadol) | 1 | 0 | 307 | 0 | 100.0% | 100.0% | 100.0% | 100.0% |
| Drug | Drug-drug interaction | Drug-induced lowering of epileptic threshold (tramadol + risperidone) | 1 | 0 | 307 | 0 | 100.0% | 100.0% | 100.0% | 100.0% |
| Drug | Adverse drug events | Drug-induced Parkinson's syndrome (biperiden + haloperidol) | 1 | 0 | 307 | 0 | 100.0% | 100.0% | 100.0% | 100.0% |
| Drug | Drug-drug interaction | Drug-induced Parkinson's syndrome (haloperidol + olanzapine) | 1 | 0 | 307 | 0 | 100.0% | 100.0% | 100.0% | 100.0% |
| Drug | Adverse drug events | Drug-induced Parkinson's syndrome (olanzapine + quetiapine) | 1 | 0 | 307 | 0 | 100.0% | 100.0% | 100.0% | 100.0% |
| Drug | Untreated indication/non-conforming with guidelines | Drug-induced Parkinson's syndrome (paroxetine et trazodone) | 1 | 0 | 307 | 0 | 100.0% | 100.0% | 100.0% | 100.0% |
| Drug | Drug-drug interaction | Drug-induced QT interval prolongation (domperidone + sulfamethoxazole+ trimethoprime) | 1 | 0 | 307 | 0 | 100.0% | 100.0% | 100.0% | 100.0% |
| Drug | Drug-drug interaction | Drug-induced QT interval prolongation (escitalopram + olanzapine + metoclopramide) | 1 | 0 | 307 | 0 | 100.0% | 100.0% | 100.0% | 100.0% |
| Drug | Drug-drug interaction | Drug-induced QT interval prolongation (hydroxychloroquine + tolterodine) | 1 | 0 | 307 | 0 | 100.0% | 100.0% | 100.0% | 100.0% |
| Drug | Drug-drug interaction | Drug-induced QT interval prolongation (amiodarone + haloperidol) | 1 | 0 | 307 | 0 | 100.0% | 100.0% | 100.0% | 100.0% |
| Drug | Drug-drug interaction | Drug-induced QT interval prolongation (amiodarone + metoprolol) | 1 | 4 | 303 | 0 | 100.0% | 98.7% | 20.0% | 100.0% |
| Drug | Drug-drug interaction | Drug-induced QT interval prolongation (amitriptyline + clarithromycine + haloperidol) | 1 | 0 | 307 | 0 | 100.0% | 100.0% | 100.0% | 100.0% |
| Drug | Drug-drug interaction | Drug-induced QT interval prolongation (ciprofloxacine + promazine + sertraline) | 1 | 0 | 307 | 0 | 100.0% | 100.0% | 100.0% | 100.0% |
| Drug | Drug-drug interaction | Drug-induced QT interval prolongation (citalopram + quetiapine) | 1 | 0 | 307 | 0 | 100.0% | 100.0% | 100.0% | 100.0% |
| Drug | Drug-drug interaction | Drug-induced QT interval prolongation (clarithromycine + citalopram) | 1 | 2 | 305 | 0 | 100.0% | 99.3% | 33.3% | 100.0% |
| Drug | Adverse drug events | Drug-induced QT interval prolongation (clarithromycine + duloxetine) | 1 | 0 | 307 | 0 | 100.0% | 100.0% | 100.0% | 100.0% |
| Drug | Drug-drug interaction | Drug-induced QT interval prolongation (clarithromycine + methadone + sulfamethoxazole (SMZ) + trimethoprime (TM)) | 1 | 0 | 307 | 0 | 100.0% | 100.0% | 100.0% | 100.0% |
| Drug | Adverse drug events | Drug-induced QT interval prolongation (clarithromycine + moxifloxacine + quetiapine) | 0 | 0 | 307 | 1 | 0.0% | 100.0% | 0.0% | 99.7% |
| Drug | Drug-drug interaction | Drug-induced QT interval prolongation (clotiapine + levofloxacine) | 1 | 0 | 307 | 0 | 100.0% | 100.0% | 100.0% | 100.0% |
| Drug | Drug-drug interaction | Drug-induced QT interval prolongation (escitalopram + domperidone + trazodone + ) | 1 | 0 | 307 | 0 | 100.0% | 100.0% | 100.0% | 100.0% |
| Drug | Drug-drug interaction | Drug-induced QT interval prolongation (escitalopram + quetiapine) | 1 | 2 | 305 | 0 | 100.0% | 99.3% | 33.3% | 100.0% |
| Drug | Drug-drug interaction | Drug-induced QT interval prolongation (methadone + fluoxetine) | 1 | 0 | 307 | 0 | 100.0% | 100.0% | 100.0% | 100.0% |
| Drug | Drug-drug interaction | Drug-induced QT interval prolongation (methadone + salmeterol) | 1 | 0 | 307 | 0 | 100.0% | 100.0% | 100.0% | 100.0% |
| Drug | Drug-drug interaction | Drug-induced QT interval prolongation (metoclopramide + domperidone) | 1 | 3 | 304 | 0 | 100.0% | 99.0% | 25.0% | 100.0% |
| Drug | Drug-drug interaction | Drug-induced QT interval prolongation (metoclopramide + ondansetron) | 1 | 4 | 303 | 0 | 100.0% | 98.7% | 20.0% | 100.0% |
| Drug | Drug-drug interaction | Drug-induced QT interval prolongation (olanzapine + fluconazole) | 1 | 0 | 307 | 0 | 100.0% | 100.0% | 100.0% | 100.0% |
| Drug | Drug-drug interaction | Drug-induced QT interval prolongation (ondansetron + domperidone) | 1 | 5 | 301 | 1 | 50.0% | 98.4% | 16.7% | 99.7% |
| Drug | Drug-drug interaction | Drug-induced QT interval prolongation (ondansetron + droperidol) | 1 | 2 | 305 | 0 | 100.0% | 99.3% | 33.3% | 100.0% |
| Drug | Drug-drug interaction | Drug-induced QT interval prolongation (paroxetine + trazodone + ciprofloxacine) | 1 | 0 | 307 | 0 | 100.0% | 100.0% | 100.0% | 100.0% |
| Drug | Drug-drug interaction | Drug-induced QT interval prolongation (ranolazine + venlafaxine + clarithromycine) | 1 | 0 | 307 | 0 | 100.0% | 100.0% | 100.0% | 100.0% |
| Drug | Drug-drug interaction | Drug-induced QT interval prolongation (risperidone + methadone) | 1 | 0 | 307 | 0 | 100.0% | 100.0% | 100.0% | 100.0% |
| Drug | Drug-drug interaction | Drug-induced QT interval prolongation (sertraline + clarithromycine) | 1 | 3 | 304 | 0 | 100.0% | 99.0% | 25.0% | 100.0% |
| Drug | Drug-drug interaction | Drug-induced QT interval prolongation (solifenacine + alfuzosine) | 1 | 0 | 307 | 0 | 100.0% | 100.0% | 100.0% | 100.0% |
| Drug | Drug-drug interaction | Drug-induced QT interval prolongation (tacrolimus + levofloxacine) | 1 | 0 | 307 | 0 | 100.0% | 100.0% | 100.0% | 100.0% |
| Drug | Drug-drug interaction | Drug-induced QT interval prolongation (tizanidine + domperidone) | 1 | 1 | 306 | 0 | 100.0% | 99.7% | 50.0% | 100.0% |
| Drug | Drug-drug interaction | Drug-induced QT interval prolongation (tolterodine + ciprofloxacine) | 1 | 0 | 307 | 0 | 100.0% | 100.0% | 100.0% | 100.0% |
| Drug | Drug-drug interaction | Drug-induced QT interval prolongation (venlafaxine + domperidone) | 1 | 0 | 307 | 0 | 100.0% | 100.0% | 100.0% | 100.0% |
| Drug | Drug-drug interaction | Drug-induced QT interval prolongation (venlafaxine + sulfamethoxazole + trimethoprime) | 1 | 0 | 307 | 0 | 100.0% | 100.0% | 100.0% | 100.0% |
| Drug | Drug-drug interaction | Drug-induced serotonin syndrome (metoclopramide + tramadol) | 1 | 1 | 306 | 0 | 100.0% | 99.7% | 50.0% | 100.0% |
| Drug | Drug-drug interaction | Drug-induced serotonin syndrome (mirtazapine + citalopram) | 1 | 0 | 307 | 0 | 100.0% | 100.0% | 100.0% | 100.0% |
| Drug | Drug-drug interaction | Drug-induced serotonin syndrome (tapentadol + amitriptyline) | 1 | 0 | 307 | 0 | 100.0% | 100.0% | 100.0% | 100.0% |
| Drug | Drug-drug interaction | Drug-induced serotonin syndrome (venlafaxine + tramadol) | 1 | 0 | 307 | 0 | 100.0% | 100.0% | 100.0% | 100.0% |
| Drug | Drug-drug interaction | Drug-induced serotonin syndrome(paroxetine + trazodone) | 1 | 0 | 307 | 0 | 100.0% | 100.0% | 100.0% | 100.0% |
| Drug | Drug-drug interaction | Drug-induced serotonin syndrome(sertraline + tramadol) | 1 | 0 | 307 | 0 | 100.0% | 100.0% | 100.0% | 100.0% |
| Drug | Underdosage | Inappropriate daily intake (aciclovir, local) | 1 | 1 | 306 | 0 | 100.0% | 99.7% | 50.0% | 100.0% |
| Drug | Overdosage | Inappropriate daily intake (clemastine) | 1 | 6 | 301 | 0 | 100.0% | 98.0% | 14.3% | 100.0% |
| Drug | Inappropriate time or frequency of administration | Inappropriate daily intake (dexamethasone) | 1 | 7 | 300 | 0 | 100.0% | 97.7% | 12.5% | 100.0% |
| Drug | Inappropriate time or frequency of administration | Inappropriate daily intake (metoprolol succinate) | 1 | 13 | 294 | 0 | 100.0% | 95.8% | 7.1% | 100.0% |
| Drug | Underdosage | Inappropriate daily intake (morphine) | 1 | 55 | 252 | 0 | 100.0% | 82.1% | 1.8% | 100.0% |
| Drug | Underdosage | Inappropriate daily intake (nicorandil) | 1 | 1 | 306 | 0 | 100.0% | 99.7% | 50.0% | 100.0% |
| Drug | Underdosage | Inappropriate daily intake (oxybutinine) | 1 | 0 | 307 | 0 | 100.0% | 100.0% | 100.0% | 100.0% |
| Drug | Inappropriate time or frequency of administration | Inappropriate daily intake (paracetamol) | 1 | 1 | 305 | 1 | 50.0% | 99.7% | 50.0% | 99.7% |
| Drug | Inappropriate time or frequency of administration | Inappropriate daily intake (paroxetine) | 1 | 0 | 307 | 0 | 100.0% | 100.0% | 100.0% | 100.0% |
| Drug | Untreated indication/non-conforming with guidelines | Inappropriate daily intake (ropinirole) | 1 | 0 | 307 | 0 | 100.0% | 100.0% | 100.0% | 100.0% |
| Drug | Underdosage | Inappropriate daily intake (spironolactone) | 1 | 1 | 306 | 0 | 100.0% | 99.7% | 50.0% | 100.0% |
| Drug | Inappropriate time or frequency of administration | Inappropriate daily intake (torasemide) | 1 | 7 | 300 | 0 | 100.0% | 97.7% | 12.5% | 100.0% |
| Drug | Underdosage | Inappropriate dosage (amiodarone) | 1 | 0 | 307 | 0 | 100.0% | 100.0% | 100.0% | 100.0% |
| Drug | Underdosage | Inappropriate dosage (amiodarone) | 1 | 0 | 307 | 0 | 100.0% | 100.0% | 100.0% | 100.0% |
| Drug | Inappropriate time or frequency of administration | Inappropriate dosage (betahistine) | 2 | 1 | 305 | 0 | 100.0% | 99.7% | 66.7% | 100.0% |
| Drug | Underdosage | Inappropriate dosage (calcitriol) | 1 | 0 | 307 | 0 | 100.0% | 100.0% | 100.0% | 100.0% |
| Drug | Underdosage | Inappropriate dosage (escitalopram) | 1 | 2 | 305 | 0 | 100.0% | 99.3% | 33.3% | 100.0% |
| Drug | Inappropriate time or frequency of administration | Inappropriate dosage (esomeprazole) | 2 | 24 | 282 | 0 | 100.0% | 92.2% | 7.7% | 100.0% |
| Drug | Untreated indication/non-conforming with guidelines | Inappropriate dosage (esomeprazole) | 2 | 24 | 282 | 0 | 100.0% | 92.2% | 7.7% | 100.0% |
| Drug | Inappropriate treatment duration | Inappropriate dosage (fosfomycine) | 1 | 0 | 307 | 0 | 100.0% | 100.0% | 100.0% | 100.0% |
| Drug | Underdosage | Inappropriate dosage (levetiracetam) | 1 | 1 | 306 | 0 | 100.0% | 99.7% | 50.0% | 100.0% |
| Drug | Inappropriate time or frequency of administration | Inappropriate dosage (metolazone) | 1 | 0 | 307 | 0 | 100.0% | 100.0% | 100.0% | 100.0% |
| Drug | Overdosage | Inappropriate dosage (mirtazapine) | 1 | 0 | 307 | 0 | 100.0% | 100.0% | 100.0% | 100.0% |
| Drug | Underdosage | Inappropriate dosage (nystatine) | 1 | 0 | 307 | 0 | 100.0% | 100.0% | 100.0% | 100.0% |
| Drug | Underdosage | Inappropriate dosage (oxycodone + naloxone) | 1 | 0 | 307 | 0 | 100.0% | 100.0% | 100.0% | 100.0% |
| Drug | Inadequate dosage for physiological state | Inappropriate dosage (paracetamol) | 1 | 16 | 289 | 2 | 33.3% | 94.8% | 5.9% | 99.3% |
| Drug | Inappropriate route of administration or galenic formulation | Inappropriate dosage (salmeterol) | 1 | 0 | 307 | 0 | 100.0% | 100.0% | 100.0% | 100.0% |
| Drug | Inappropriate time or frequency of administration | Inappropriate dosage (setraline) | 1 | 0 | 307 | 0 | 100.0% | 100.0% | 100.0% | 100.0% |
| Drug | Inappropriate route of administration or galenic formulation | Inappropriate galenic form (short acting instead of long acting metoprolol) | 1 | 2 | 304 | 1 | 50.0% | 99.3% | 33.3% | 99.7% |
| Drug | Adverse drug events | Inappropriate monitoring for benefit/risk balance assessment (fluticasone) | 1 | 0 | 307 | 0 | 100.0% | 100.0% | 100.0% | 100.0% |
| Drug | Adverse drug events | Inappropriate monitoring for benefit/risk balance assessment (glycopyrronium + indacaterol) | 1 | 0 | 307 | 0 | 100.0% | 100.0% | 100.0% | 100.0% |
| Drug | Inappropriate route of administration or galenic formulation | Inappropriate route of administration (thiamine) | 14 | 12 | 275 | 7 | 66.7% | 95.8% | 53.8% | 97.5% |
| Drug | Untreated indication/non-conforming with guidelines | Non-compliance with guidelines (no adrenergic agonist + montelukast) | 1 | 2 | 305 | 0 | 100.0% | 99.3% | 33.3% | 100.0% |
| Drug | Inappropriate route of administration or galenic formulation | Parenteral administration while the oral route is available (amoxicillin + clavulanic acid) | 1 | 61 | 246 | 0 | 100.0% | 80.1% | 1.6% | 100.0% |
| Drug | Inappropriate route of administration or galenic formulation | Parenteral administration while the oral route is available (metoclopramide) | 2 | 17 | 289 | 0 | 100.0% | 94.4% | 10.5% | 100.0% |
| Drug | Inappropriate route of administration or galenic formulation | Parenteral administration while the oral route is available (morphine) | 2 | 33 | 273 | 0 | 100.0% | 89.2% | 5.7% | 100.0% |
| Drug | Inappropriate route of administration or galenic formulation | Parenteral administration while the oral route is available (ondansetron) | 1 | 7 | 300 | 0 | 100.0% | 97.7% | 12.5% | 100.0% |
| Drug | Inappropriate route of administration or galenic formulation | Parenteral administration while the oral route is available (paracetamol) | 7 | 24 | 277 | 0 | 100.0% | 92.0% | 22.6% | 100.0% |
| Drug | Inappropriate route of administration or galenic formulation | Parenteral administration while the oral route is available (potassium) | 0 | 17 | 290 | 1 | 0.0% | 94.5% | 0.0% | 99.7% |
| Drug | Untreated indication/non-conforming with guidelines | Risk of bleeding (acencoumarol + acetylsalicilic acid) | 2 | 12 | 294 | 0 | 100.0% | 96.1% | 14.3% | 100.0% |
| Drug | Adverse drug events | Risk of bleeding (acenocoumarol + acetylsalicilic acid + paroxetine) | 1 | 0 | 307 | 0 | 100.0% | 100.0% | 100.0% | 100.0% |
| Drug | Drug-drug interaction | Risk of bleeding (acenocoumarol + acetylsalicylic acid) | 1 | 13 | 294 | 0 | 100.0% | 95.8% | 7.1% | 100.0% |
| Drug | Drug-drug interaction | Risk of bleeding (acetylsalicilic acid + acenocoumarol) | 1 | 13 | 294 | 0 | 100.0% | 95.8% | 7.1% | 100.0% |
| Drug | Drug-drug interaction | Risk of bleeding (acetylsalicilic acid + duloxetine + rivaroxaban) | 1 | 0 | 307 | 0 | 100.0% | 100.0% | 100.0% | 100.0% |
| Drug | Drug-drug interaction | Risk of bleeding (acetylsalicilic acid + paroxetine + enoxaparine) | 1 | 1 | 306 | 0 | 100.0% | 99.7% | 50.0% | 100.0% |
| Drug | Drug-drug interaction | Risk of bleeding (acetylsalicilic acid + prednisone + citalopram) | 1 | 0 | 307 | 0 | 100.0% | 100.0% | 100.0% | 100.0% |
| Drug | Drug-drug interaction | Risk of bleeding (apixaban + clarithromycine) | 1 | 0 | 307 | 0 | 100.0% | 100.0% | 100.0% | 100.0% |
| Drug | Adverse drug events | Risk of bleeding (citalopram + clopidogrel + enoxaparine) | 1 | 0 | 307 | 0 | 100.0% | 100.0% | 100.0% | 100.0% |
| Drug | Adverse drug events | Risk of bleeding (enoxaparin + citalopram) | 1 | 2 | 305 | 0 | 100.0% | 99.3% | 33.3% | 100.0% |
| Drug | Drug-drug interaction | Risk of bleeding (enoxaparine + citalopram + ibuprofène) | 1 | 0 | 307 | 0 | 100.0% | 100.0% | 100.0% | 100.0% |
| Drug | Drug-drug interaction | Risk of bleeding (rivaroxaban + duloxetine + nifedipine) | 1 | 0 | 307 | 0 | 100.0% | 100.0% | 100.0% | 100.0% |
| Drug | Drug-drug interaction | Risk of bleeding (rivaroxaban + escitalopram) | 1 | 0 | 307 | 0 | 100.0% | 100.0% | 100.0% | 100.0% |
| Drug | Drug-drug interaction | Risk of bleeding (rivaroxaban + ibuprofène) | 1 | 1 | 306 | 0 | 100.0% | 99.7% | 50.0% | 100.0% |
| Drug | Drug-drug interaction | Risk of bleeding (rivaroxaban + simvastatine) | 1 | 0 | 307 | 0 | 100.0% | 100.0% | 100.0% | 100.0% |
| Drug | Adverse drug events | Unfavourable benefit-risk balance (statin + fibrate) | 1 | 0 | 306 | 1 | 50.0% | 100.0% | 100.0% | 99.7% |
| Drug-Demographic | Inadequate dosage for physiological state | Benefit/risk balance not favourable given the age (digoxin) | 1 | 1 | 306 | 0 | 100.0% | 99.7% | 50.0% | 100.0% |
| Drug-Demographic | Adverse drug events | Benefit/risk balance not favourable given the age (estradiol) | 1 | 0 | 307 | 0 | 100.0% | 100.0% | 100.0% | 100.0% |
| Drug-Demographic | Untreated indication/non-conforming with guidelines | Benefit/risk balance not favourable given the age (hydroxyzine) | 1 | 0 | 307 | 0 | 100.0% | 100.0% | 100.0% | 100.0% |
| Drug-Demographic | Inadequate dosage for physiological state | Benefit/risk balance not favourable given the age (paracetamol) | 1 | 17 | 290 | 0 | 100.0% | 94.5% | 5.6% | 100.0% |
| Drug-Demographic | Inadequate dosage for physiological state | Benefit/risk balance not favourable given the weight (paracetamol) | 2 | 10 | 293 | 3 | 40.0% | 96.7% | 16.7% | 99.0% |
| Drug-Demographic | Adverse drug events | Contraindication/precaution of use (ezetimibe + simvastatine + advanced age) | 1 | 0 | 307 | 0 | 100.0% | 100.0% | 100.0% | 100.0% |
| Drug-Lab | Adverse drug events | Contraindication/precaution of use (amoxicillin and clavulanic acid + hepathotoxicity) | 1 | 18 | 289 | 0 | 100.0% | 94.1% | 5.3% | 100.0% |
| Drug-Lab | Adverse drug events | Contraindication/precaution of use (duloxetine + hyponatremia) | 1 | 1 | 306 | 0 | 100.0% | 99.7% | 50.0% | 100.0% |
| Drug-Lab | Adverse drug events | Contraindication/precaution of use (esomeprazole + vitamin B12 deficiency) | 1 | 13 | 293 | 1 | 50.0% | 95.8% | 7.1% | 99.7% |
| Drug-Lab | Underdosage | Contraindication/precaution of use (levothyroxine + high TSH levels) | 1 | 1 | 306 | 0 | 100.0% | 99.7% | 50.0% | 100.0% |
| Drug-Lab | Adverse drug events | Contraindication/precaution of use (montelukast + hepathotoxicity) | 1 | 2 | 305 | 0 | 100.0% | 99.3% | 33.3% | 100.0% |
| Drug-Lab | Adverse drug events | Contraindication/precaution of use (nonfractionated heparin + no acute renal failure) | 3 | 33 | 272 | 0 | 100.0% | 89.2% | 8.3% | 100.0% |
| Drug-Lab | Inadequate dosage for physiological state | Contraindication/precaution of use (paracetamol dosage not adjusted to hepathotoxicity) | 1 | 119 | 186 | 2 | 33.3% | 61.0% | 0.8% | 98.9% |
| Drug-Lab | Overprescription—duplicate therapy | Contraindication/precaution of use (potassium chloride + hyperkaliemia) | 2 | 15 | 290 | 1 | 66.7% | 95.1% | 11.8% | 99.7% |
| Drug-Lab | Overprescription—duplicate therapy | Contraindication/precaution of use (potassium chloride + normokaliema) | 1 | 28 | 279 | 0 | 100.0% | 90.9% | 3.4% | 100.0% |
| Drug-Lab | Overprescription—duplicate therapy | Contraindication/precaution of use (sodium bicarbonate + alkalosis) | 1 | 15 | 292 | 0 | 100.0% | 95.1% | 6.3% | 100.0% |
| Drug-Lab | Adverse drug events | Contraindication/precaution of use (tizanidine + hepathotoxicity) | 1 | 1 | 306 | 0 | 100.0% | 99.7% | 50.0% | 100.0% |
| Drug-Lab | Adverse drug events | Contraindication/precaution of use (torasemide + hypokaliemia) | 1 | 56 | 251 | 0 | 100.0% | 81.8% | 1.8% | 100.0% |
| Drug-Lab | Adverse drug events | Contraindication/precaution of use (venlafaxine + hyponatremia) | 1 | 3 | 304 | 0 | 100.0% | 99.0% | 25.0% | 100.0% |
| Drug-Lab | Inadequate dosage for physiological state | Drug accumulation exposig to various side effects (ciprofloxacine not adjusted to renal impairement) | 1 | 2 | 305 | 0 | 100.0% | 99.3% | 33.3% | 100.0% |
| Drug-Lab | Inappropriate treatment duration | Drug accumulation exposig to various side effects (folic acid + supranormal blood folate levels) | 2 | 3 | 303 | 0 | 100.0% | 99.0% | 40.0% | 100.0% |
| Drug-Lab | Inadequate dosage for physiological state | Drug accumulation exposig to various side effects (gabapentine not adjusted to renal impairement) | 1 | 1 | 306 | 0 | 100.0% | 99.7% | 50.0% | 100.0% |
| Drug-Lab | Adverse drug events | Drug accumulation exposig to various side effects (ibuprofen + acute kidney failure) | 0 | 1 | 306 | 1 | 0.0% | 99.7% | 0.0% | 99.7% |
| Drug-Lab | Inadequate dosage for physiological state | Drug accumulation exposig to various side effects (imipenem and cilastatin not adjusted to (mild) renal impairement) | 1 | 10 | 297 | 0 | 100.0% | 96.7% | 9.1% | 100.0% |
| Drug-Lab | Inadequate dosage for physiological state | Drug accumulation exposig to various side effects (imipenem and cilastatin not adjusted to (severe) renal impairement) | 1 | 6 | 301 | 0 | 100.0% | 98.0% | 14.3% | 100.0% |
| Drug-Lab | Inadequate dosage for physiological state | Drug accumulation exposig to various side effects (losartan + acute kidney failure) | 0 | 1 | 306 | 1 | 0.0% | 99.7% | 0.0% | 99.7% |
| Drug-Lab | Inadequate dosage for physiological state | Drug accumulation exposig to various side effects (morphine dosage not adjusted to renal impairement) | 1 | 0 | 307 | 0 | 100.0% | 100.0% | 100.0% | 100.0% |
| Drug-Lab | Underdosage | Drug accumulation exposig to various side effects (parenteral amoxicilline + clavulanic acid IV not adjusted to renal impairement) | 1 | 0 | 307 | 0 | 100.0% | 100.0% | 100.0% | 100.0% |
| Drug-Lab | Inadequate dosage for physiological state | Drug accumulation exposig to various side effects (piperacillin and tazobactam not adjusted to renal impairement) | 1 | 15 | 292 | 0 | 100.0% | 95.1% | 6.3% | 100.0% |
| Drug-Lab | Inadequate dosage for physiological state | Drug accumulation exposig to various side effects (sitagliptine not adjusted to renal impairement) | 1 | 1 | 306 | 0 | 100.0% | 99.7% | 50.0% | 100.0% |
| Drug-Lab | Overdosage | Drug accumulation exposig to various side effects (supratherapeutic blood level of levetiracetam) | 1 | 0 | 307 | 0 | 100.0% | 100.0% | 100.0% | 100.0% |
| Drug-Lab | Adverse drug events | Drug accumulation exposig to various side effects (trospium + acute kidney failure) | 0 | 0 | 307 | 1 | 0.0% | 100.0% | 0.0% | 99.7% |
| Drug-Lab | Adverse drug events | Drug accumulation exposig to various side effects (valsartan + acute kidney failure) | 1 | 0 | 307 | 0 | 100.0% | 100.0% | 100.0% | 100.0% |
| Drug-Lab | Adverse drug events | Hepatic side effect (riluzole + abnormal liver biochemicals tests) | 1 | 0 | 307 | 0 | 100.0% | 100.0% | 100.0% | 100.0% |
| Drug-Lab | Adverse drug events | Inappropriate monitoring for benefit/risk balance assessment (amiodaron) | 1 | 8 | 299 | 0 | 100.0% | 97.4% | 11.1% | 100.0% |
| Drug-Lab | Inappropriate monitoring | Inappropriate monitoring for benefit/risk balance assessment (clarithromycine) | 2 | 23 | 283 | 0 | 100.0% | 92.5% | 8.0% | 100.0% |
| Drug-Lab | Inappropriate monitoring | Inappropriate monitoring for benefit/risk balance assessment (dapaglifozine) | 1 | 0 | 307 | 0 | 100.0% | 100.0% | 100.0% | 100.0% |
| Drug-Lab | Inappropriate monitoring | Inappropriate monitoring for benefit/risk balance assessment (glibenclamide) | 1 | 0 | 307 | 0 | 100.0% | 100.0% | 100.0% | 100.0% |
| Drug-Lab | Inappropriate monitoring | Inappropriate monitoring for benefit/risk balance assessment (gliclazide) | 2 | 4 | 302 | 0 | 100.0% | 98.7% | 33.3% | 100.0% |
| Drug-Lab | Inappropriate monitoring | Inappropriate monitoring for benefit/risk balance assessment (glimepiride or metformine or sitagliptine) | 12 | 25 | 265 | 6 | 66.7% | 91.4% | 32.4% | 97.8% |
| Drug-Lab | Inappropriate monitoring | Inappropriate monitoring for benefit/risk balance assessment (insulin aspart) | 7 | 41 | 257 | 3 | 70.0% | 86.2% | 14.6% | 98.8% |
| Drug-Lab | Inappropriate monitoring | Inappropriate monitoring for benefit/risk balance assessment (insulin glargin) | 1 | 1 | 304 | 2 | 33.3% | 99.7% | 50.0% | 99.3% |
| Drug-Lab | Inappropriate monitoring | Inappropriate monitoring for benefit/risk balance assessment (insulin protamine) | 6 | 10 | 292 | 0 | 100.0% | 96.7% | 37.5% | 100.0% |
| Drug-Lab | Inappropriate monitoring | Inappropriate monitoring for benefit/risk balance assessment (levothyroxine) | 1 | 14 | 293 | 0 | 100.0% | 95.4% | 6.7% | 100.0% |
| Drug-Lab | Inappropriate monitoring | Inappropriate monitoring for benefit/risk balance assessment (linagliptin) | 2 | 0 | 305 | 1 | 66.7% | 100.0% | 100.0% | 99.7% |
| Drug-Lab | Inappropriate monitoring | Inappropriate monitoring for benefit/risk balance assessment (polystyrene sulfonate) | 0 | 11 | 296 | 1 | 0.0% | 96.4% | 0.0% | 99.7% |
| Drug-Lab | Adverse drug events | Inappropriate monitoring for benefit/risk balance assessment (prednisone) | 3 | 48 | 257 | 0 | 100.0% | 84.3% | 5.9% | 100.0% |
| Drug-Lab | Adverse drug events | Inappropriate monitoring for benefit/risk balance assessment (valproic acid) | 1 | 4 | 303 | 0 | 100.0% | 98.7% | 20.0% | 100.0% |
| Drug-Lab | Adverse drug events | Non-compliance with guidelines (electrolytes with carbohydrates + normoglycemia) | 1 | 0 | 307 | 0 | 100.0% | 100.0% | 100.0% | 100.0% |
| Drug-Lab | Inadequate dosage for physiological state | Risk of bleeding (apixaban + acute kidney failure) | 1 | 0 | 307 | 0 | 100.0% | 100.0% | 100.0% | 100.0% |
| Drug-Lab | Inadequate dosage for physiological state | Risk of bleeding (rivaroxaban + acute kidney failure) | 1 | 0 | 307 | 0 | 100.0% | 100.0% | 100.0% | 100.0% |
| Drug-Lab | Untreated indication/non-conforming with guidelines | Untreated condition (no calcium carbonate with hyperphosphatemia) | 3 | 30 | 275 | 0 | 100.0% | 90.2% | 9.1% | 100.0% |
| Drug-Lab | Untreated indication/non-conforming with guidelines | Untreated condition (no folic acid with folate deficiency) | 0 | 18 | 289 | 1 | 0.0% | 94.1% | 0.0% | 99.7% |
| Drug-Lab | Untreated indication/non-conforming with guidelines | Untreated condition (no iron with iron deficiency anaemia) | 1 | 21 | 286 | 0 | 100.0% | 93.2% | 4.5% | 100.0% |
| Drug-Lab | Untreated indication/non-conforming with guidelines | Untreated condition (no iron with iron deficiency) | 1 | 36 | 271 | 0 | 100.0% | 88.3% | 2.7% | 100.0% |
| Drug-Lab | Untreated indication/non-conforming with guidelines | Untreated condition (no potassium supplementation with hypokaliemia) | 4 | 178 | 126 | 0 | 100.0% | 41.4% | 2.2% | 100.0% |
| Drug-Lab | Untreated indication/non-conforming with guidelines | Untreated condition (no vitamin D3 with hypovitaminosis D) | 5 | 29 | 261 | 13 | 27.8% | 90.0% | 14.7% | 95.3% |
| Drug-Problem | Adverse drug events | Anticholinegic side effects (amitriptyline + at-risk condition) | 1 | 0 | 307 | 0 | 100.0% | 100.0% | 100.0% | 100.0% |
| Drug-Problem | Adverse drug events | Anticholinegic side effects (darifenacine + at-risk condition) | 1 | 0 | 307 | 0 | 100.0% | 100.0% | 100.0% | 100.0% |
| Drug-Problem | Adverse drug events | Anticholinegic side effects (fexofenadine + at-risk condition) | 1 | 0 | 307 | 0 | 100.0% | 100.0% | 100.0% | 100.0% |
| Drug-Problem | Adverse drug events | Anticholinegic side effects (galantamine + at-risk condition) | 1 | 0 | 307 | 0 | 100.0% | 100.0% | 100.0% | 100.0% |
| Drug-Problem | Adverse drug events | Anticholinegic side effects (hydroxyzine + at-risk condition) | 1 | 0 | 307 | 0 | 100.0% | 100.0% | 100.0% | 100.0% |
| Drug-Problem | Adverse drug events | Anticholinegic side effects (solifenacine + at-risk condition) | 2 | 0 | 306 | 0 | 100.0% | 100.0% | 100.0% | 100.0% |
| Drug-Problem | Adverse drug events | Anticholinegic side effects (trospium + at-risk condition) | 1 | 0 | 307 | 0 | 100.0% | 100.0% | 100.0% | 100.0% |
| Drug-Problem | Adverse drug events | Central nervous system side effects (sertraline + at-risk condition) | 1 | 0 | 307 | 0 | 100.0% | 100.0% | 100.0% | 100.0% |
| Drug-Problem | Untreated indication/non-conforming with guidelines | Contraindication/precaution of use (acetylsalicilic acid + gastritis) | 1 | 1 | 306 | 0 | 100.0% | 99.7% | 50.0% | 100.0% |
| Drug-Problem | Untreated indication/non-conforming with guidelines | Contraindication/precaution of use (acetylsalicilic acid + ishemic heart disease) | 1 | 3 | 304 | 0 | 100.0% | 99.0% | 25.0% | 100.0% |
| Drug-Problem | Overdosage | Contraindication/precaution of use (amoxicilline and clavulanic acid dosage not adjusted to indication) | 1 | 1 | 306 | 0 | 100.0% | 99.7% | 50.0% | 100.0% |
| Drug-Problem | Untreated indication/non-conforming with guidelines | Contraindication/precaution of use (bromazepam or zolpidem + confusion) | 1 | 1 | 306 | 0 | 100.0% | 99.7% | 50.0% | 100.0% |
| Drug-Problem | Adverse drug events | Contraindication/precaution of use (cetirizine + at-risk condition) | 1 | 1 | 306 | 0 | 100.0% | 99.7% | 50.0% | 100.0% |
| Drug-Problem | Untreated indication/non-conforming with guidelines | Contraindication/precaution of use (clomethiazole + confusion) | 1 | 1 | 306 | 0 | 100.0% | 99.7% | 50.0% | 100.0% |
| Drug-Problem | Untreated indication/non-conforming with guidelines | Contraindication/precaution of use (cyproterone + at-risk condition) | 1 | 0 | 307 | 0 | 100.0% | 100.0% | 100.0% | 100.0% |
| Drug-Problem | Inadequate dosage for physiological state | Contraindication/precaution of use (duloxetine + at-risk condition) | 1 | 0 | 307 | 0 | 100.0% | 100.0% | 100.0% | 100.0% |
| Drug-Problem | Inappropriate time or frequency of administration | Contraindication/precaution of use (enalapril dosage not adjusted to indication) | 1 | 3 | 304 | 0 | 100.0% | 99.0% | 25.0% | 100.0% |
| Drug-Problem | Adverse drug events | Contraindication/precaution of use (ibuprofen + at-risk condition) | 1 | 6 | 301 | 0 | 100.0% | 98.0% | 14.3% | 100.0% |
| Drug-Problem | Untreated indication/non-conforming with guidelines | Contraindication/precaution of use (lorazepam + cognitive disorders) | 1 | 4 | 303 | 0 | 100.0% | 98.7% | 20.0% | 100.0% |
| Drug-Problem | Overprescription—duplicate therapy | Contraindication/precaution of use (nitroglycerine + at-risk condition) | 1 | 21 | 286 | 0 | 100.0% | 93.2% | 4.5% | 100.0% |
| Drug-Problem | Untreated indication/non-conforming with guidelines | Contraindication/precaution of use (olanzapine + at-risk condition) | 1 | 7 | 300 | 0 | 100.0% | 97.7% | 12.5% | 100.0% |
| Drug-Problem | Inadequate dosage for physiological state | Contraindication/precaution of use (paracetamol dosage not adjusted to hepatic steathosis) | 0 | 3 | 304 | 1 | 0.0% | 99.0% | 0.0% | 99.7% |
| Drug-Problem | Inadequate dosage for physiological state | Contraindication/precaution of use (paracetamol dosage not adjusted to liver alcoholism) | 2 | 13 | 289 | 4 | 33.3% | 95.7% | 13.3% | 98.6% |
| Drug-Problem | Inadequate dosage for physiological state | Contraindication/precaution of use (paracetamol dosage not adjusted to liver cancer) | 1 | 7 | 285 | 15 | 6.3% | 97.6% | 12.5% | 95.0% |
| Drug-Problem | Inadequate dosage for physiological state | Contraindication/precaution of use (paracetamol dosage not adjusted to undernutrition and renal failure) | 1 | 0 | 307 | 0 | 100.0% | 100.0% | 100.0% | 100.0% |
| Drug-Problem | Untreated indication/non-conforming with guidelines | Contraindication/precaution of use (systemic antibiotic + viral bronchitis) | 1 | 42 | 265 | 0 | 100.0% | 86.3% | 2.3% | 100.0% |
| Drug-Problem | Untreated indication/non-conforming with guidelines | Contraindication/precaution of use (ticagrelor + at-risk condition) | 1 | 2 | 305 | 0 | 100.0% | 99.3% | 33.3% | 100.0% |
| Drug-Problem | Adverse drug events | Contraindication/precaution of use (tramadol + at-risk condition) | 1 | 1 | 306 | 0 | 100.0% | 99.7% | 50.0% | 100.0% |
| Drug-Problem | Adverse drug events | Contraindication/precaution of use (zolpidem + at-risk condition) | 1 | 2 | 305 | 0 | 100.0% | 99.3% | 33.3% | 100.0% |
| Drug-Problem | Inadequate dosage for physiological state | Drug accumulation exposig to various side effects (zolpidem dosage not adjusted to renal impairement) | 1 | 11 | 296 | 0 | 100.0% | 96.4% | 8.3% | 100.0% |
| Drug-Problem | Untreated indication/non-conforming with guidelines | Non-compliance with guidelines (no ACE inhibitor + ACE inhibitor indication) | 2 | 12 | 294 | 0 | 100.0% | 96.1% | 14.3% | 100.0% |
| Drug-Problem | Untreated indication/non-conforming with guidelines | Non-compliance with guidelines (no acetylsaliclic acid + acetylsaliclic acid indication) | 2 | 0 | 306 | 0 | 100.0% | 100.0% | 100.0% | 100.0% |
| Drug-Problem | Untreated indication/non-conforming with guidelines | Non-compliance with guidelines (no amiodarone + amiodarone indication) | 1 | 3 | 304 | 0 | 100.0% | 99.0% | 25.0% | 100.0% |
| Drug-Problem | Untreated indication/non-conforming with guidelines | Non-compliance with guidelines (no angiotensin II receptor blockers or ACE inhibitor + heart failure) | 1 | 18 | 288 | 1 | 50.0% | 94.1% | 5.3% | 99.7% |
| Drug-Problem | Untreated indication/non-conforming with guidelines | Non-compliance with guidelines (no angiotensin II receptor blockers or ACE inhibitor +angiotensin II receptor blockers or ACE inhibitor, except heart failure) | 2 | 67 | 238 | 1 | 66.7% | 78.0% | 2.9% | 99.6% |
| Drug-Problem | Untreated indication/non-conforming with guidelines | Non-compliance with guidelines (no anticoagulants + anticoagulants indication) | 1 | 12 | 295 | 0 | 100.0% | 96.1% | 7.7% | 100.0% |
| Drug-Problem | Untreated indication/non-conforming with guidelines | Non-compliance with guidelines (no antiretroviral + antiretroviral indication) | 1 | 3 | 304 | 0 | 100.0% | 99.0% | 25.0% | 100.0% |
| Drug-Problem | Untreated indication/non-conforming with guidelines | Non-compliance with guidelines (no calcium + vitamin D3 + calcium + vitamin D3 indication) | 1 | 0 | 307 | 0 | 100.0% | 100.0% | 100.0% | 100.0% |
| Drug-Problem | Untreated indication/non-conforming with guidelines | Non-compliance with guidelines (no fluticasone with salmeterol + fluticasone with salmeterol indication) | 1 | 7 | 300 | 0 | 100.0% | 97.7% | 12.5% | 100.0% |
| Drug-Problem | Untreated indication/non-conforming with guidelines | Non-compliance with guidelines (no metformine + metformine indication) | 1 | 39 | 268 | 0 | 100.0% | 87.3% | 2.5% | 100.0% |
| Drug-Problem | Untreated indication/non-conforming with guidelines | Non-compliance with guidelines (no metoprolol + metoprolol indication) | 1 | 10 | 297 | 0 | 100.0% | 96.7% | 9.1% | 100.0% |
| Drug-Problem | Untreated indication/non-conforming with guidelines | Non-compliance with guidelines (no mirtazapine + mirtazapine indication) | 1 | 0 | 307 | 0 | 100.0% | 100.0% | 100.0% | 100.0% |
| Drug-Problem | Untreated indication/non-conforming with guidelines | Non-compliance with guidelines (no propranolol + propranolol indication) | 1 | 2 | 305 | 0 | 100.0% | 99.3% | 33.3% | 100.0% |
| Drug-Problem | Untreated indication/non-conforming with guidelines | Non-compliance with guidelines (no statin + statin indication) | 9 | 105 | 191 | 3 | 75.0% | 64.5% | 7.9% | 98.5% |
| Drug-Problem | Untreated indication/non-conforming with guidelines | Non-compliance with guidelines (no valproic acid + valproic acid indication) | 1 | 2 | 305 | 0 | 100.0% | 99.3% | 33.3% | 100.0% |
| Drug-Problem | Untreated indication/non-conforming with guidelines | Non-compliance with guidelines (no Vitamin B1 + Vitamin B1 indication) | 1 | 7 | 299 | 1 | 50.0% | 97.7% | 12.5% | 99.7% |
| Drug-Problem | Overprescription—duplicate therapy | Treatment without clear indication exposing to a side effect (acetylcysteine) | 1 | 0 | 307 | 0 | 100.0% | 100.0% | 100.0% | 100.0% |
| Drug-Problem | Overprescription—duplicate therapy | Treatment without clear indication exposing to a side effect (acetylsalicilic acid) | 6 | 69 | 230 | 3 | 66.7% | 76.9% | 8.0% | 98.7% |
| Drug-Problem | Overprescription—duplicate therapy | Treatment without clear indication exposing to a side effect (aciclovir) | 1 | 1 | 306 | 0 | 100.0% | 99.7% | 50.0% | 100.0% |
| Drug-Problem | Overprescription—duplicate therapy | Treatment without clear indication exposing to a side effect (alendronate) | 1 | 1 | 306 | 0 | 100.0% | 99.7% | 50.0% | 100.0% |
| Drug-Problem | Overprescription—duplicate therapy | Treatment without clear indication exposing to a side effect (allopurinol) | 4 | 5 | 298 | 1 | 80.0% | 98.3% | 44.4% | 99.7% |
| Drug-Problem | Overprescription—duplicate therapy | Treatment without clear indication exposing to a side effect (amoxicilline + clavulanic acid) | 2 | 85 | 221 | 0 | 100.0% | 72.2% | 2.3% | 100.0% |
| Drug-Problem | Overprescription—duplicate therapy | Treatment without clear indication exposing to a side effect (atorvastatine) | 2 | 37 | 269 | 0 | 100.0% | 87.9% | 5.1% | 100.0% |
| Drug-Problem | Overprescription—duplicate therapy | Treatment without clear indication exposing to a side effect (betahistine) | 1 | 2 | 305 | 0 | 100.0% | 99.3% | 33.3% | 100.0% |
| Drug-Problem | Overprescription—duplicate therapy | Treatment without clear indication exposing to a side effect (calcium and vitamin D3) | 1 | 47 | 260 | 0 | 100.0% | 84.7% | 2.1% | 100.0% |
| Drug-Problem | Overprescription—duplicate therapy | Treatment without clear indication exposing to a side effect (calcium or vitamin D3) | 2 | 9 | 288 | 9 | 18.2% | 97.0% | 18.2% | 97.0% |
| Drug-Problem | Overprescription—duplicate therapy | Treatment without clear indication exposing to a side effect (cetirizine) | 2 | 11 | 294 | 1 | 66.7% | 96.4% | 15.4% | 99.7% |
| Drug-Problem | Overprescription—duplicate therapy | Treatment without clear indication exposing to a side effect (cetylpiridine) | 5 | 5 | 298 | 0 | 100.0% | 98.3% | 50.0% | 100.0% |
| Drug-Problem | Overprescription—duplicate therapy | Treatment without clear indication exposing to a side effect (citalopram) | 1 | 7 | 299 | 1 | 50.0% | 97.7% | 12.5% | 99.7% |
| Drug-Problem | Overprescription—duplicate therapy | Treatment without clear indication exposing to a side effect (clopidogrel) | 1 | 14 | 293 | 0 | 100.0% | 95.4% | 6.7% | 100.0% |
| Drug-Problem | Overprescription—duplicate therapy | Treatment without clear indication exposing to a side effect (diltiazem) | 1 | 0 | 307 | 0 | 100.0% | 100.0% | 100.0% | 100.0% |
| Drug-Problem | Overprescription—duplicate therapy | Treatment without clear indication exposing to a side effect (diltiazem) | 1 | 0 | 307 | 0 | 100.0% | 100.0% | 100.0% | 100.0% |
| Drug-Problem | Overprescription—duplicate therapy | Treatment without clear indication exposing to a side effect (domperidone) | 1 | 28 | 279 | 0 | 100.0% | 90.9% | 3.4% | 100.0% |
| Drug-Problem | Overprescription—duplicate therapy | Treatment without clear indication exposing to a side effect (duloxetine) | 2 | 3 | 303 | 0 | 100.0% | 99.0% | 40.0% | 100.0% |
| Drug-Problem | Overprescription—duplicate therapy | Treatment without clear indication exposing to a side effect (dutastride + tamsulosine) | 1 | 2 | 305 | 0 | 100.0% | 99.3% | 33.3% | 100.0% |
| Drug-Problem | Overprescription—duplicate therapy | Treatment without clear indication exposing to a side effect (enoxaparine) | 5 | 126 | 174 | 3 | 62.5% | 58.0% | 3.8% | 98.3% |
| Drug-Problem | Overprescription—duplicate therapy | Treatment without clear indication exposing to a side effect (enoxaparine) | 5 | 126 | 174 | 3 | 62.5% | 58.0% | 3.8% | 98.3% |
| Drug-Problem | Overprescription—duplicate therapy | Treatment without clear indication exposing to a side effect (escitalopram) | 1 | 10 | 295 | 2 | 33.3% | 96.7% | 9.1% | 99.3% |
| Drug-Problem | Overprescription—duplicate therapy | Treatment without clear indication exposing to a side effect (esomeprazole) | 54 | 79 | 155 | 20 | 73.0% | 66.2% | 40.6% | 88.6% |
| Drug-Problem | Overprescription—duplicate therapy | Treatment without clear indication exposing to a side effect (estradiol + norethisterone) | 1 | 0 | 306 | 1 | 50.0% | 100.0% | 100.0% | 99.7% |
| Drug-Problem | Overprescription—duplicate therapy | Treatment without clear indication exposing to a side effect (fexofenadine) | 1 | 0 | 307 | 0 | 100.0% | 100.0% | 100.0% | 100.0% |
| Drug-Problem | Overprescription—duplicate therapy | Treatment without clear indication exposing to a side effect (finasteride) | 1 | 1 | 306 | 0 | 100.0% | 99.7% | 50.0% | 100.0% |
| Drug-Problem | Overprescription—duplicate therapy | Treatment without clear indication exposing to a side effect (fluconazole) | 1 | 6 | 301 | 0 | 100.0% | 98.0% | 14.3% | 100.0% |
| Drug-Problem | Overprescription—duplicate therapy | Treatment without clear indication exposing to a side effect (fluoxetine) | 1 | 2 | 305 | 0 | 100.0% | 99.3% | 33.3% | 100.0% |
| Drug-Problem | Overprescription—duplicate therapy | Treatment without clear indication exposing to a side effect (folic acid) | 4 | 13 | 289 | 2 | 66.7% | 95.7% | 23.5% | 99.3% |
| Drug-Problem | Overprescription—duplicate therapy | Treatment without clear indication exposing to a side effect (fondaparinux) | 1 | 19 | 287 | 1 | 50.0% | 93.8% | 5.0% | 99.7% |
| Drug-Problem | Overprescription—duplicate therapy | Treatment without clear indication exposing to a side effect (iron supplementation) | 1 | 0 | 307 | 0 | 100.0% | 100.0% | 100.0% | 100.0% |
| Drug-Problem | Overprescription—duplicate therapy | Treatment without clear indication exposing to a side effect (latanoprost) | 1 | 1 | 306 | 0 | 100.0% | 99.7% | 50.0% | 100.0% |
| Drug-Problem | Overprescription—duplicate therapy | Treatment without clear indication exposing to a side effect (levetiracetam) | 1 | 5 | 302 | 0 | 100.0% | 98.4% | 16.7% | 100.0% |
| Drug-Problem | Overprescription—duplicate therapy | Treatment without clear indication exposing to a side effect (levothyroxine) | 1 | 14 | 292 | 1 | 50.0% | 95.4% | 6.7% | 99.7% |
| Drug-Problem | Overprescription—duplicate therapy | Treatment without clear indication exposing to a side effect (lisinopril) | 1 | 10 | 297 | 0 | 100.0% | 96.7% | 9.1% | 100.0% |
| Drug-Problem | Overprescription—duplicate therapy | Treatment without clear indication exposing to a side effect (lorazepam) | 1 | 50 | 257 | 0 | 100.0% | 83.7% | 2.0% | 100.0% |
| Drug-Problem | Overprescription—duplicate therapy | Treatment without clear indication exposing to a side effect (magaldrate) | 1 | 16 | 291 | 0 | 100.0% | 94.8% | 5.9% | 100.0% |
| Drug-Problem | Overprescription—duplicate therapy | Treatment without clear indication exposing to a side effect (mebevirine) | 1 | 6 | 301 | 0 | 100.0% | 98.0% | 14.3% | 100.0% |
| Drug-Problem | Overprescription—duplicate therapy | Treatment without clear indication exposing to a side effect (metformine) | 1 | 7 | 300 | 0 | 100.0% | 97.7% | 12.5% | 100.0% |
| Drug-Problem | Overprescription—duplicate therapy | Treatment without clear indication exposing to a side effect (metoprolol) | 1 | 14 | 292 | 1 | 50.0% | 95.4% | 6.7% | 99.7% |
| Drug-Problem | Overprescription—duplicate therapy | Treatment without clear indication exposing to a side effect (mirabegron) | 1 | 0 | 307 | 0 | 100.0% | 100.0% | 100.0% | 100.0% |
| Drug-Problem | Overprescription—duplicate therapy | Treatment without clear indication exposing to a side effect (mirtazapine) | 2 | 5 | 298 | 3 | 40.0% | 98.3% | 28.6% | 99.0% |
| Drug-Problem | Overprescription—duplicate therapy | Treatment without clear indication exposing to a side effect (nitroglycerine) | 1 | 20 | 287 | 0 | 100.0% | 93.5% | 4.8% | 100.0% |
| Drug-Problem | Overprescription—duplicate therapy | Treatment without clear indication exposing to a side effect (nystatine) | 1 | 9 | 298 | 0 | 100.0% | 97.1% | 10.0% | 100.0% |
| Drug-Problem | Overprescription—duplicate therapy | Treatment without clear indication exposing to a side effect (pancreatin) | 1 | 5 | 302 | 0 | 100.0% | 98.4% | 16.7% | 100.0% |
| Drug-Problem | Overprescription—duplicate therapy | Treatment without clear indication exposing to a side effect (paroxetine) | 3 | 4 | 301 | 0 | 100.0% | 98.7% | 42.9% | 100.0% |
| Drug-Problem | Overprescription—duplicate therapy | Treatment without clear indication exposing to a side effect (picosulfate) | 1 | 97 | 210 | 0 | 100.0% | 68.4% | 1.0% | 100.0% |
| Drug-Problem | Overprescription—duplicate therapy | Treatment without clear indication exposing to a side effect (pramipexole) | 1 | 2 | 305 | 0 | 100.0% | 99.3% | 33.3% | 100.0% |
| Drug-Problem | Overprescription—duplicate therapy | Treatment without clear indication exposing to a side effect (prednisone) | 1 | 55 | 252 | 0 | 100.0% | 82.1% | 1.8% | 100.0% |
| Drug-Problem | Overprescription—duplicate therapy | Treatment without clear indication exposing to a side effect (pregabalin) | 2 | 10 | 296 | 0 | 100.0% | 96.7% | 16.7% | 100.0% |
| Drug-Problem | Overprescription—duplicate therapy | Treatment without clear indication exposing to a side effect (primidone) | 2 | 0 | 306 | 0 | 100.0% | 100.0% | 100.0% | 100.0% |
| Drug-Problem | Overprescription—duplicate therapy | Treatment without clear indication exposing to a side effect (quetiapine) | 1 | 11 | 295 | 1 | 50.0% | 96.4% | 8.3% | 99.7% |
| Drug-Problem | Overprescription—duplicate therapy | Treatment without clear indication exposing to a side effect (ranitidine) | 1 | 1 | 305 | 1 | 50.0% | 99.7% | 50.0% | 99.7% |
| Drug-Problem | Overprescription—duplicate therapy | Treatment without clear indication exposing to a side effect (risedronate) | 1 | 0 | 307 | 0 | 100.0% | 100.0% | 100.0% | 100.0% |
| Drug-Problem | Overprescription—duplicate therapy | Treatment without clear indication exposing to a side effect (risperidone) | 1 | 4 | 303 | 0 | 100.0% | 98.7% | 20.0% | 100.0% |
| Drug-Problem | Overprescription—duplicate therapy | Treatment without clear indication exposing to a side effect (salmeterol + fluticasone) | 1 | 27 | 280 | 0 | 100.0% | 91.2% | 3.6% | 100.0% |
| Drug-Problem | Overprescription—duplicate therapy | Treatment without clear indication exposing to a side effect (sertraline) | 3 | 5 | 300 | 0 | 100.0% | 98.4% | 37.5% | 100.0% |
| Drug-Problem | Overprescription—duplicate therapy | Treatment without clear indication exposing to a side effect (solifenacine) | 1 | 2 | 305 | 0 | 100.0% | 99.3% | 33.3% | 100.0% |
| Drug-Problem | Overprescription—duplicate therapy | Treatment without clear indication exposing to a side effect (tamsulosine) | 3 | 8 | 295 | 2 | 60.0% | 97.4% | 27.3% | 99.3% |
| Drug-Problem | Overprescription—duplicate therapy | Treatment without clear indication exposing to a side effect (tetrazosine) | 1 | 0 | 307 | 0 | 100.0% | 100.0% | 100.0% | 100.0% |
| Drug-Problem | Overprescription—duplicate therapy | Treatment without clear indication exposing to a side effect (tizanidine) | 1 | 5 | 302 | 0 | 100.0% | 98.4% | 16.7% | 100.0% |
| Drug-Problem | Overprescription—duplicate therapy | Treatment without clear indication exposing to a side effect (trazodone) | 1 | 7 | 300 | 0 | 100.0% | 97.7% | 12.5% | 100.0% |
| Drug-Problem | Overprescription—duplicate therapy | Treatment without clear indication exposing to a side effect (valproic acid) | 2 | 3 | 303 | 0 | 100.0% | 99.0% | 40.0% | 100.0% |
| Drug-Problem | Overprescription—duplicate therapy | Treatment without clear indication exposing to a side effect (venlafaxine) | 1 | 9 | 298 | 0 | 100.0% | 97.1% | 10.0% | 100.0% |
| Drug-Problem | Overprescription—duplicate therapy | Treatment without clear indication exposing to a side effect (Vitamin B) | 2 | 28 | 275 | 3 | 40.0% | 90.8% | 6.7% | 98.9% |
| Drug-Problem | Adverse drug events | Untreated condition (no metoprolol + at-risk condition) | 1 | 0 | 307 | 0 | 100.0% | 100.0% | 100.0% | 100.0% |
| Drug-Vital signs | Overdosage | Benefit/risk balance not favourable given the age (atorvastatine high dosage) | 1 | 2 | 305 | 0 | 100.0% | 99.3% | 33.3% | 100.0% |
| Drug-Vital signs | Adverse drug events | Contraindication/precaution of use (duloxetine and arterial hypertension) | 1 | 2 | 305 | 0 | 100.0% | 99.3% | 33.3% | 100.0% |
| Drug-Vital signs | Adverse drug events | Contraindication/precaution of use (oxycodone without laxatives and constipation) | 0 | 0 | 307 | 1 | 0.0% | 100.0% | 0.0% | 99.7% |
| Drug-Vital signs | Adverse drug events | Contraindication/precaution of use (tapentadol without laxatives and constipation) | 0 | 1 | 306 | 1 | 0.0% | 99.7% | 0.0% | 99.7% |
| Drug-Vital signs | Adverse drug events | Contraindication/precaution of use (tramadol without laxatives and constipation) | 1 | 9 | 296 | 2 | 33.3% | 97.0% | 10.0% | 99.3% |
| Drug-Vital signs | Inappropriate monitoring | Inappropriate monitoring for benefit/risk balance assessment (oxazepam) | 1 | 44 | 263 | 0 | 100.0% | 85.7% | 2.2% | 100.0% |
| Drug-Vital signs | Overprescription—duplicate therapy | Treatment without clear indication exposing to a side effect (morphine) | 1 | 31 | 276 | 0 | 100.0% | 89.9% | 3.1% | 100.0% |
| Drug-Vital signs | Overprescription—duplicate therapy | Treatment without clear indication exposing to a side effect (oxycodone) | 1 | 2 | 305 | 0 | 100.0% | 99.3% | 33.3% | 100.0% |
| Drug-Vital signs | Underdosage | Treatment without clear indication exposing to a side effect (paracetamol + codeine) | 1 | 2 | 305 | 0 | 100.0% | 99.3% | 33.3% | 100.0% |
| Drug-Vital signs | Overprescription—duplicate therapy | Treatment without clear indication exposing to a side effect (paracetamol) | 1 | 85 | 222 | 0 | 100.0% | 72.3% | 1.2% | 100.0% |
| Drug-Vital signs | Inappropriate route of administration or galenic formulation | Untreated condition (no non-opioid or weak opioid analgesic + pain) | 1 | 189 | 118 | 0 | 100.0% | 38.4% | 0.5% | 100.0% |
| Complex queries | Untreated indication/non-conforming with guidelines | Contraindication/precaution of use (angiotensin II receptor blockers or ACE inhibitor + at-risk condition) | 2 | 52 | 253 | 1 | 66.7% | 83.0% | 3.7% | 99.6% |
| Complex queries | Adverse drug events | Contraindication/precaution of use (buprenorphine or methadone or morphine without laxatives and constipation) | 6 | 19 | 278 | 5 | 54.5% | 93.6% | 24.0% | 98.2% |
| Complex queries | Adverse drug events | Contraindication/precaution of use (hydromorphone + at-risk condition) | 1 | 0 | 307 | 0 | 100.0% | 100.0% | 100.0% | 100.0% |
| Complex queries | Adverse drug events | Contraindication/precaution of use (prednisone + at-risk condition) | 1 | 50 | 257 | 0 | 100.0% | 83.7% | 2.0% | 100.0% |
| Complex queries | Adverse drug events | Drug-induced central nervous system depression (clemastine + at-risk condition) | 1 | 0 | 307 | 0 | 100.0% | 100.0% | 100.0% | 100.0% |
| Complex queries | Inadequate dosage for physiological state | Inappropriate dosage (over-dosed digoxin + at-risk condition) | 0 | 3 | 304 | 1 | 0.0% | 99.0% | 0.0% | 99.7% |
| Complex queries | Inadequate dosage for physiological state | Inappropriate dosage (over-dosed paracetamol + at-risk condition) | 0 | 19 | 287 | 2 | 0.0% | 93.8% | 0.0% | 99.3% |
| Complex queries | Underdosage | Inappropriate dosage (under-dosed spironolactone + conditions requiring high-dosage) | 2 | 6 | 299 | 1 | 66.7% | 98.0% | 25.0% | 99.7% |
| Complex queries | Underdosage | Inappropriate dosage (under-dosed statin + conditions requiring high-dosage) | 0 | 4 | 303 | 1 | 0.0% | 98.7% | 0.0% | 99.7% |
| Complex queries | Inappropriate monitoring | Inappropriate monitoring for benefit/risk balance assessment (enoxaparin) | 1 | 10 | 297 | 0 | 100.0% | 96.7% | 9.1% | 100.0% |
| Complex queries | Untreated indication/non-conforming with guidelines | Inappropriate monitoring for benefit/risk balance assessment (lactic acid producing organisms) | 4 | 2 | 302 | 0 | 100.0% | 99.3% | 66.7% | 100.0% |
| Complex queries | Adverse drug events | Inappropriate monitoring for benefit/risk balance assessment (memantine) | 1 | 0 | 307 | 0 | 100.0% | 100.0% | 100.0% | 100.0% |
| Complex queries | Untreated indication/non-conforming with guidelines | Inappropriate monitoring for benefit/risk balance assessment (ranolazine) | 1 | 0 | 307 | 0 | 100.0% | 100.0% | 100.0% | 100.0% |
| Complex queries | Overprescription—duplicate therapy | Treatment without clear indication exposing to a side effect (magnesium chloride) | 15 | 32 | 260 | 1 | 93.8% | 89.0% | 31.9% | 99.6% |
| Complex queries | Overdosage | Treatment without clear indication exposing to a side effect (statin at high dosage) | 1 | 13 | 294 | 0 | 100.0% | 95.8% | 7.1% | 100.0% |
| Complex queries | Untreated indication/non-conforming with guidelines | Untreated condition (no heparin + heparin indication) | 0 | 8 | 299 | 1 | 0.0% | 97.4% | 0.0% | 99.7% |
| Complex queries | Untreated indication/non-conforming with guidelines | Untreated condition (no magnesium chloride + magnesium chloride inidcation) | 1 | 9 | 298 | 0 | 100.0% | 97.1% | 10.0% | 100.0% |
| Complex queries | Untreated indication/non-conforming with guidelines | Untreated condition (no spironolactone + spironolactone indication) | 1 | 14 | 293 | 0 | 100.0% | 95.4% | 6.7% | 100.0% |
